# Identification and Reconstitution of the First Two Enzymatic Steps for the Biosynthesis of Bioactive Meroterpenoids from *Hericium erinaceus* (Lion’s Mane Mushroom)

**DOI:** 10.3390/molecules29235576

**Published:** 2024-11-26

**Authors:** Riccardo Iacovelli, Fons Poon, Kristina Haslinger

**Affiliations:** Department of Chemical and Pharmaceutical Biology, Groningen Research Institute of Pharmacy, University of Groningen, 9713 AV Groningen, The Netherlands

**Keywords:** natural products, meroterpenoids, lion’s mane mushroom, *Hericium erinaceus*, pathway reconstitution, heterologous expression

## Abstract

*Hericium erinaceus* (Lion’s Mane mushroom) is widely consumed for its numerous reported benefits for brain health. A growing body of evidence suggests that these benefits are likely attributable to aromatic compounds contained in its fruiting bodies, including the meroterpenoids hericenones. Here, we report the identification and reconstitution of the first two steps of the biosynthetic pathway of hericenones via heterologous expression of the polyketide synthase HerA and the carboxylic acid reductase HerB in *Aspergillus oryzae*. Furthermore, we investigated a putative prenyltransferase that might be responsible for the following biosynthetic step. Ongoing efforts to reconstitute the full pathway will enable large-scale production of hericenones and other meroterpenoids in heterologous hosts.

## 1. Introduction

**Figure 1:**
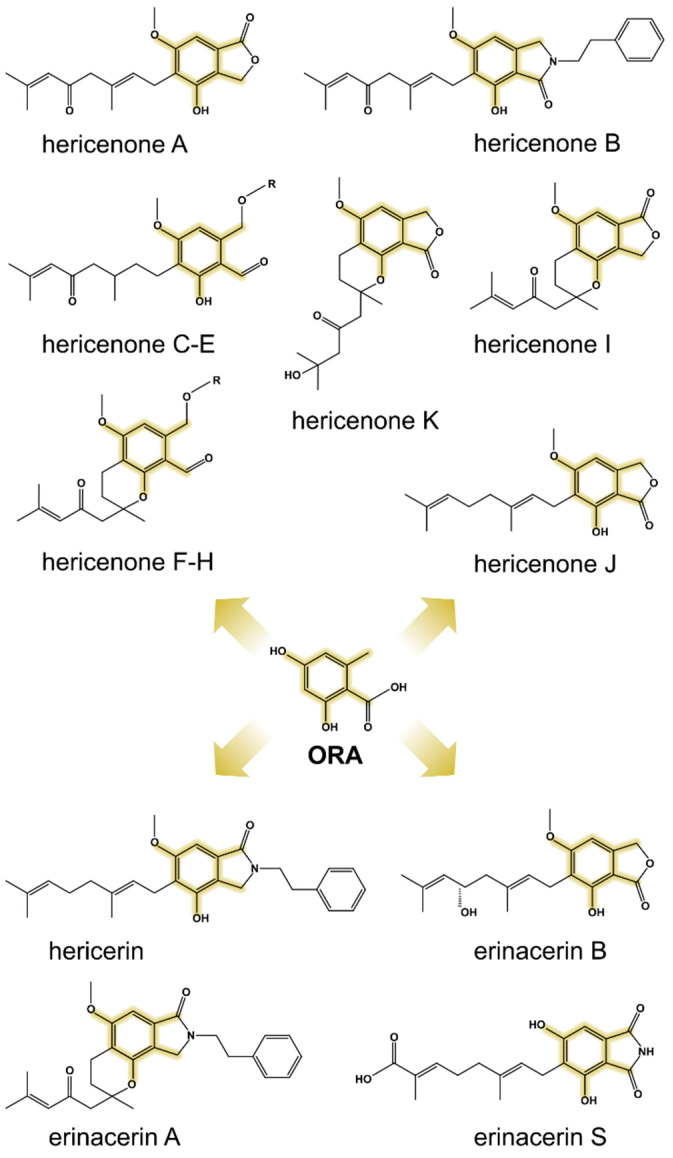
Revised structures [18] of some ORA-derived meroterpenoids from *H. erinaceus*. The orsellinic acid core is highlighted in yellow. For hericenones, R = palmitoyl (C, F), stearoyl (D, G), or linoleoyl (E, H).

Fungal natural products (NPs)—also known as secondary metabolites—are an important source for the discovery of bioactive compounds with pharmaceutical applications [1,2]. Traditionally, research in the field has focused on NPs from ascomycetes, particularly molds from the genera *Aspergillus* and *Penicillium*, given that these are genetically tractable and generally easy to cultivate in the laboratory [3,4,5,6,7]. Recent advances in -omics sciences allowed researchers to expand the scope of NP discovery to other groups of fungi, including edible mushrooms and medicinal mushrooms belonging to the phylum *Basidiomycota* [8,9,10]. Among these, the Lion’s Mane fungus—*Hericium erinaceus*—has gained considerable attention due to its reported health-promoting effects, attributable to the antioxidative, anti-inflammatory, and immunostimulating properties of its bioactive constituents [11,12]. These mainly include complex polysaccharides [13,14], the diterpenes erinacines, and orsellinic acid (ORA)-derived meroterpenoids (hericenones, hericerins, and erinacerins) (Figure 1), mainly produced in the fruiting bodies [12,15,16,17,18]. These meroterpenoids belong to a broader class of compounds that exhibit potent biological activities with important applications in medicine [19,20]. For example, mycophenolic acid produced by *Penicillium brevicompactum* has long been a widely prescribed immunosuppressant medication [21,22]; cannabidiol extracted from *Cannabis* plants was recently approved for the treatment of seizures [23,24]; and daurichromenic acid isolated from the plant *Rhododendron dauricum* is being extensively studied for its remarkable anti-HIV activity [25,26].

Recent research has shown that the meroterpenoids from *H. erinaceus* exhibit neuroprotective and neuro-regenerative effects on isolated neuronal cells and in mouse models [15,17,27,28,29,30], making these molecules interesting candidates for developing potential treatments for neurodegenerative diseases, such as dementia and Alzheimer’s, and for neuronal injuries. Although hericenones were first isolated from *H. erinaceus* more than 30 years ago [31], the biosynthetic machinery responsible for the synthesis of meroterpenoids in *H. erinaceus* remains yet to be identified. Knowing the responsible enzymes would provide access to enhanced compound production—for example, by heterologous expression of the biosynthetic genes in model fungi or by metabolic engineering of the native producer.

In this study, we identified a candidate biosynthetic gene cluster (BGC) for the biosynthesis of ORA-derived meroterpenoids in the genome of *H. erinaceus*. Next, we expressed the two core enzymes in a heterologous host and confirmed the successful production of orsellinic aldehyde—the central meroterpenoid scaffold. Our results provide the first direct link between these valuable natural products and the underlying biosynthetic route, paving the way toward complete characterization of the pathway and reconstitution in heterologous hosts for compound production.

## 2. Results and Discussion

### 2.1. The Genome of Hericium erinaceus Encodes a Putative Hericenone BGC

**Figure 2:**
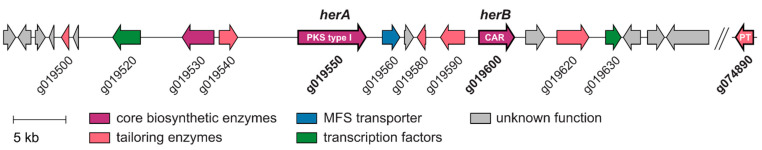
Putative hericenones BGC from *H. erinaceus*. The genes subjects of this study are highlighted. The putative prenyltransferase-encoding gene g074890 is depicted separated by a double slash symbol since it is not part of the cluster, and it was identified via a homology-based search strategy. The putative functions of the BGC genes are shown in Table 1.

Orsellinic acid—the core structure shared by hericenones, hericerin, and erinacerins—is one of the simplest aromatic polyketides, and is biosynthesized by type III PKS in plants and by type I PKS in bacteria and fungi [32]. In several meroterpenoids of *H. erinaceus*, the carboxylic acid moiety is reduced to an aldehyde, indicating that an aromatic carboxylic acid reductase (CAR) might also be involved in the biosynthesis [33]. Meroterpenoid-producing BGCs that encode these enzymes have been recently identified in the ascomycete filamentous fungi *Stachybotrys bisbyi* [34] and *Acremonium egyptiacum* [35]. In both cases, a type I PKS (ORA synthase), a prenyltransferase (PT) with farnesyltransferase activity, and a CAR (orsellinic acid reductase) are involved in the first three biosynthetic steps that yield the intermediate ilicicolin B (also called LL-Z 1272β), a farnesylated orsellinic aldehyde. Therefore, we set out to identify a similar BGC in *H. erinaceus*. For that, we first retrieved the genome assembly of *H. erinaceus* 0605 from the NCBI database [36] and directly submitted it to the webtool fungiSMASH v7.0 [37] for BGC prediction. Out of the twelve predicted BGCs (Additional File S2), we identified only one BGC encoding a type I non-reducing PKS and two putative NRPS-like enzymes, the enzyme family that CARs belong to (Figure 2).

Interestingly, previous research has shown that the expression of the PKS gene is upregulated in the meroterpenoid-producing fruiting bodies of the lion’s mane compared to the mycelium, which supports a link between this BGC and the meroterpenoids [38]. Unfortunately, upon closer inspection of the BGC, we did not find a prenyltransferase-encoding gene. It is common in basidiomycete fungi that enzymes underlying a specific biosynthetic pathway are encoded on different loci in the genome [10]. Thus, we re-annotated the *H. erinaceus* 0605 genome using GenSAS v6.0 [39] (Additional File S3) and performed a homology-based search within its proteome using the amino acid sequences of prenyltransferases StbC and AscA as queries. We identified g074890—on a different contig—as the most likely PT-encoding candidate gene (Tables S1 and S2, Additional File S2).

### 2.2. Overexpression of herA and herB in A. oryzae Results in the Production of Orsellinic Aldehyde

**Figure 3:**
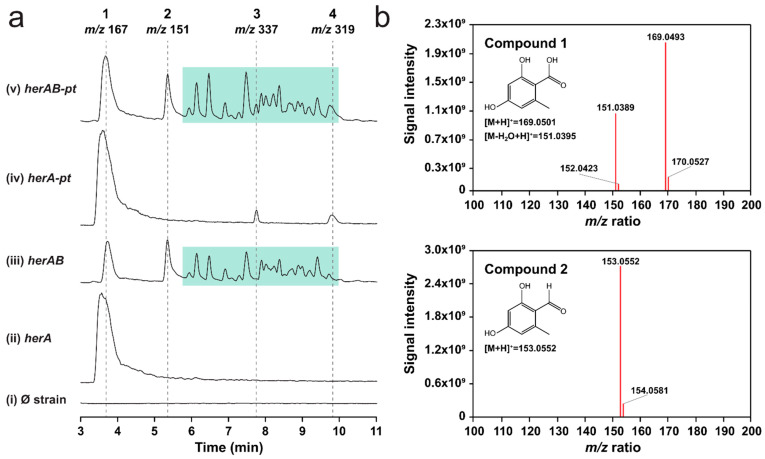
Heterologous production of orsellinic acid and orsellinic aldehyde in *A. oryzae* NSAR1. (**a**) Extracted ion chromatograms (ESI−) of fungal extracts displaying *m/z* 167 (**1**), 151 (**2**), 337 (**3**), 319 (**4**), and a series of *m/z* values ranging between 259 and 983 detected only in *herA* and *herB* co-expression strains (Additional File S3), highlighted by a green-shaded box. (i) *A. oryzae* NSAR1, background strain; (ii)–(v) *her* expression strains. To optimize the size of the figure, g074890 is abbreviated as *pt* in the strain labels. (**b**) HRMS2 spectra of orsellinic acid (**1**) and orsellinic aldehyde (**2**) in the fungal extracts. Spectra were recorded in positive ionization mode.

We then proceeded with the in vivo characterization efforts. First, we cloned *herA* into the pTYargB plasmid [40], under control of the *amyB* promoter, and expressed it in the host *A. oryzae* NSAR1 [41]. Following cultivation on induction medium, we analyzed extra- and intra-cellular metabolites via liquid chromatography–diode-array detection–mass spectrometry (HPLC-DAD-MS) analysis and detected a prominent new peak (**1**) in the fungal extracts with an *m/z* of 167 (ESI−), corresponding to the expected value for orsellinic acid (Figure 3a, Figure S1). Analysis of the UV and HRMS2 spectra confirmed this observation (Figure 3b, Figure S2). Around the same time, Han and coworkers [42] also reported the successful expression of *herA* in *A. oryzae*, which was confirmed to be an orsellinic acid synthase. Interestingly, the sequence they published is 155 nt shorter than the one we obtained via PCR. When we examined the sequence of *herA* that was predicted by GenSAS, we realized that this was a perfect match with the published sequence (Table S1). Thus, we hypothesize that the 155 nt—and in general, the full genome—were inadequately annotated by the basic annotation tool of fungiSMASH, as opposed to the more accurate GenSAS pipeline that we applied. This prompted us to re-run the BGC prediction, this time supplying the annotation file we generated. With this addition, antiSMASH predicted more BGCs on the genome (20 vs. 12, Additional File S2), and several features within the putative hericenones BGC showed significant differences, including redefined gene boundaries and the prediction of a previously undetected transcription factor (Figure S3). The revised BGC is shown in Figure 2, while the predicted functions of the genes are listed in Table 1. Two striking differences were the predicted size and genomic coordinates of NRPS-like–encoding gene 39—now labeled as g019600 (Figure S3, Table S3).

**Figure 4:**
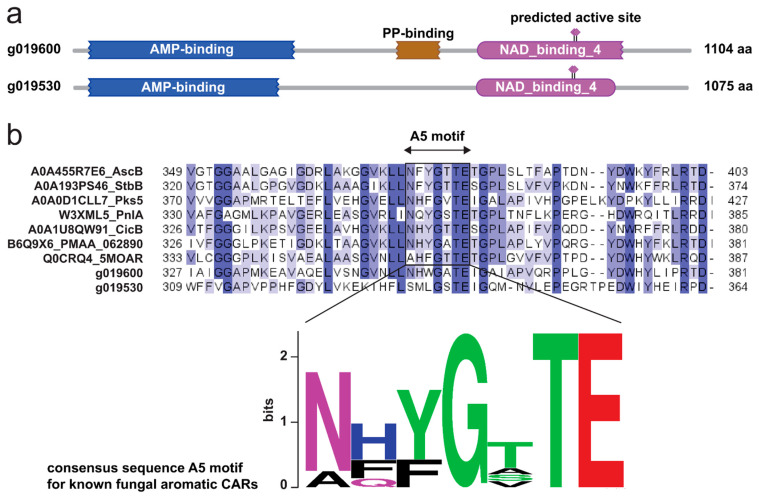
Criteria for selection of the CAR gene. (**a**) domain prediction performed with the HMMER webserver [43,44] indicated that g019600 has all domains needed for CAR activity, whereas g019530 seems to be missing the phosphopantheteine attachment site required for the activated substrate to be transported to the reductase domain. (**b**) MSA analysis of the two candidate genes and known fungal aromatic CARs from previous studies [33] shows a high degree of conservation within the key A5 motif of the adenylation domain for g019600, but not for g019530. The sequence logo for the A5 motif was generated with the WebLogo tool [45].

To prioritize our cloning efforts, we performed bioinformatic analyses on the two NRPS-like–encoding genes. We found that g019600 was likely the functional CAR because it shows the overall domain arrangement typical of this family of enzymes, including an adenylation domain (A), a peptidyl carrier protein (PCP) domain, and a terminal NAD-dependent reductase domain (R) [33]. Furthermore, g019600 shows a conserved sequence in the A5 structural motif—crucial for substrate recognition—found in the N-terminal subdomain of the A domains of aromatic fungal CARs. In contrast, our bioinformatic analysis showed that g019530 lacks the PCP domain and the conserved sequence in the A5 motif, suggesting that it might have a completely different function that remains to be identified (Figure 4). Thus, we transformed the *herA* expression strain with the pTYadeA [40] plasmid harboring g019600 (*herB*). The resulting co-expression strain showed slightly stunted growth and a different pigmentation compared to the background and *herA* expression strains (Figure S4). When we analyzed the fungal extracts via HPLC-DAD-MS, we observed consumption of orsellinic acid and detected a second peak (**2**) with an *m/z* value of 151 (ESI−), corresponding to the expected value for orsellinic aldehyde (Figure 3a, Figure S1). HRMS-MS analysis confirmed the chemical identity of the compound (Figure 3b).

Interestingly, we also detected several other peaks, with *m/z* values ranging from 259 to 983 (ESI−) (green box in Figure 3a, Figure S1). Fungal strains that express *herB* alone do not produce either orsellinic aldehyde or these metabolites (Figure S1). From the HRMS-MS data (Additional File S3), it appears that these compounds are derivatives of orsellinic acid or aldehyde, since their HRMS2 spectra show low molecular weight fragments in the *m/z* value range of 120–170, which are compatible with (sub)structures of orsellinic acid. When we inspected their combined spectra (Figure S5), we found that they showed repeated mass differences of 136.0530 and 43.9900 Da, corresponding to C_8_H_8_O_2_ (orsellinic acid minus 2 oxygen atoms) and CO_2_, respectively. We hypothesize that orsellinic aldehyde can react with orsellinic acid leading to the formation of polymers. The 43.9900 Da difference can be explained by spontaneous decarboxylation, which occurs easily when orsellinic acid is in solution. Despite these observations, we cannot confirm our hypothesis without further characterization by NMR. Furthermore, we do not know whether polymerization happens in vivo or after metabolite extraction. Nevertheless, our results confirm that HerB is the functional CAR within the BGC, and that it can act together with HerA to produce orsellinic aldehyde, the core structure of the meroterpenoids from *H. erinaceus*.

### 2.3. Overexpression of Gene g074890 Yields Two New Compounds That May Be Prenylated Variants of Orsellinic Acid

Lastly, we cloned and expressed the putative PT encoded by g074890 in both the *herA* and *herAB* expression strains. Following LC-MS analysis of the extracts, we detected two new peaks (**3** and **4**), with respective *m/z* values of 337 and 319 (ESI−) (Figure 3a). These compounds are only formed when *herA* and g074890 are co-expressed (Figure 3a, Figure S1), independently of *herB* co-expression, indicating that they derive from orsellinic acid. Unfortunately, neither **3** nor **4** show the expected *m/z* value of geranylated orsellinc acid—304 (ESI−)—expected to be produced by the PT. Based on preliminary HRMS-MS analysis, however, we hypothesize that compound **3** might indeed be a geranylated variant of orsellinic acid, albeit with two additional hydroxyl groups (Figure S6). Compound **4** shows similarities with **3** in its HRMS2 spectrum (Figure S7) and, based on its *m/z* value of 319 (ESI−), we could hypothesize that it is a geranylated variant of orsellinic acid with one additional hydroxyl group. Unfortunately, we do not see the corresponding ion in positive ionization mode (*m/z* 321), which makes the hypothesis more challenging. NMR analysis of isolated compounds **3** and **4** is needed to confirm these observations. However, given their limited abundance in the fungal extracts, scaling up the cultivation and developing a suitable purification procedure are required.

## 3. Conclusions

In conclusion, we employed bioinformatic analysis and heterologous expression to identify the first two steps of the biosynthetic pathway of meroterpenoids in *H. erinaceus*. Namely, we successfully expressed the non-reducing PKS HerA and the CAR HerB, necessary to deliver orsellinic aldehyde, which is the central core structure of hericenones, erinacerins, and hericerin. With these results, we can link for the first time the biosynthesis of these compounds to a candidate BGC. We also identified and expressed a putative geranyltransferase located outside the BGC which could be involved in the third biosynthetic step, although its function needs to be confirmed. It is also possible that another—yet to be identified—geranyltransferase is responsible for the decoration of orsellinic aldehyde, or that g074890 is active on a later pathway intermediate. Lastly, several genes found in the cluster are predicted to encode tailoring enzymes—including a flavoprotein, a monooxygenase, an SDR, and an aldehyde dehydrogenase—which are likely required to functionalize the orsellinic core structure and generate the different meroterpenoids. It is not excluded that more enzymes encoded by genes outside the BGC participate in the biosynthetic pathway. Ongoing efforts to reconstitute the full BGC will be instrumental in deciphering additional biosynthetic steps.

Overall, our work offers early insights into the biosynthesis of bioactive meroterpenoids from *H. erinaceus*, enabling future endeavors to fully elucidate the pathway and reconstitute it in heterologous hosts to produce meroterpenoids for pharmaceutical applications.

## 4. Materials and Methods

### 4.1. Bioinformatic Analyses

Structural annotation of the genome of *H. erinaceus* 0605 (NCBI acc. no. GCA_016906435.1) was performed with the Genome Sequence Annotation Server (GenSAS) v6.0 [39]. Default settings were used unless otherwise mentioned. Briefly, low-complexity regions and repeats were masked using RepeatModeler v2.0.3 and RepeatMasker v4.1.1 [46], setting the DNA source to ‘Fungi’. The masked consensus sequence was used for ab initio gene prediction using the following tools: (I) Augustus v3.4.0 [47] selecting *Coprinopsis cinerea* as a trained organism; (II) GeneMarkES v4.48 [48]. The NCBI reference transcript and protein databases for fungi were used for homology-based prediction, using the tools (III) blastn v2.12.0 [49] and (IV) DIAMOND v2.0.11 [50], respectively. Lastly, EvidenceModeler v1.1.1 [51] was used to generate the consensus model from the above-mentioned predictions, weighed as follows: (I)—five, (II)—five, (III)—ten, (IV)—ten.

Biosynthetic gene clusters in the genome of *H. erinaceus* were predicted using antiSMASH web server v7.0 [37] with default settings. The assembly (FASTA) file was used as the sole input for the first prediction, and later in combination with the annotation file (GFF3, Additional File S4) generated by GenSAS.

For the identification of putative PT-encoding genes, the sequences of StbC (UniProt acc. no. A0A193PS58) [34] and AscA (UniProt acc. no. A0A455R413) [35] were retrieved from the UniProt database [52] and submitted as search queries to phmmer (HMMER v3.3.2) [44] against the total proteome of *H. erinaceus* 0605. The cutoff was set at an E-value of 0.01 (Additional File S2). The Sequence Manipulation Suite [53] was used to calculate identity and similarity percentages between HerA, HerB, and g074890, and the corresponding homologous proteins from the *stb* and *asc* BGCs.

The HMMER v3.3.2 webtool was used to analyze the domain architecture of candidate CARs g019530 and g019600 (Figure 4). The multiple sequence alignment analysis between known fungal aromatic CARs—retrieved from the UniProt database and described previously [33]—and g019530 and g019600 was performed with MEGA 11 [54] using the MUSCLE algorithm [55] with default settings, and visualized in Jalview [56].

### 4.2. Fungal Strains

*H. erinaceus* CBS 302.89 (reisolated from an infected culture originating from Taiwan) was obtained from the Westerdijk Institute strain collection (Utrecht, The Netherlands). The fungus was routinely maintained on MEA agar (malt extract 30 g/L; peptone 5 g/L; microagar 15 g/L in ddH_2_O) at 20 °C in the dark. For extraction of genomic DNA, *H. erinaceus* was grown for 14 days in malt extract broth (as MEA, without agar) in static conditions at 20 °C in the dark. The biomass was harvested and freeze-dried, and genomic DNA was extracted with the Nucleospin Microbial DNA kit (Qiagen, Venlo, The Netherlands), as previously described for other fungi [57].

*A. oryzae* NSAR1 (ΔargB, adeA−, sC−, and niaD−) [41] was kindly provided by Prof. Jun-ichi Maruyama from the University of Tokyo, Japan. The fungus was routinely maintained on DPY agar (20 g/L glucose; 10 g/L peptone; 5 g/L yeast extract; 0.5 g/L MgSO_4_·7H_2_O; 5 g/L KH_2_PO_4_; microagar 15 g/L in ddH_2_O; pH 5.5), and cultivated on DPY-KCl agar (5 g/L glucose; 10 g/L peptone; 5 g/L yeast extract; 0.5 g/L MgSO_4_ · 7H_2_O; 5 g/L KH_2_PO_4_; 45 g/L KCl; 1 mL/L Hutner’s trace element solution; [58] microagar 15 g/L in ddH_2_O; pH 5.5) to induce sporulation. The plates were incubated at 30 °C for 5–7 days.

### 4.3. Amplification and Molecular Cloning of herA, herB, and g074890

The integrative expression vectors pTYargB-*eGFPac*, pTYadeA-*eGFPac*, and pTYsC-*eGFPac* were kindly provided by Dr. Colin Lazarus from the University of Bristol, UK. These were digested with FastDigest NotI and PacI and dephosphorylated with FastAP phosphatase (Thermo Fisher Scientific, Waltham, MA, USA). The vector fragments were then purified via gel extraction using the QIAquick Gel Extraction Kit (Qiagen, Hilden, Germany). The *herA*, *herB*, and g074890 genes were amplified from genomic DNA of *H. erinaceus* CBS 302.89 and cloned by sticky-end ligation in the inducible amyB cassette of the pTYargB, pTYadeA, and pTYsC vectors, respectively (Figure S8). PCR reactions were performed using 2x Q5 PCR master mix (New England Biolabs, Ipswich, MA, USA) and 1 µL of template (~10 ng gDNA) in a total volume of 25 µL, according to the manufacturer’s instructions. Primers and other parameters are listed in Table S4. Routine procedures were used for transforming chemically competent *E. coli* DH10β cells with the assembled constructs. Positive clones were selected on LB agar supplemented with ampicillin 100 µg/mL. Direct-colony PCR was used to pick positive transformants. The corresponding plasmids were extracted using the QIAprep Spin Miniprep Kit (Qiagen) and sent to Macrogen Europe (Amsterdam, the Netherlands) for verification by Sanger sequencing.

### 4.4. Genetic Transformation of A. oryzae NSAR1

Protoplasts of *A. oryzae* NSAR1 were obtained from spore suspensions and transformed as previously described [59]. For the expression of single genes, protoplasts were mixed with approximately 1–2 µg (max 10 µL) of pTYargB-*herA*, pTYadeA-*herB*, or pTYsC-g074890. For co-expression of multiple genes, protoplasts were prepared from the *herA* expression strain and transformed with either pTYadeA-*herB* or pTYsC-g074890 alone (double transformants), or with both plasmids (triple transformant). In all cases, the total amount of DNA used was approximately 1–2 µg (max 10 µL). Protoplasts were regenerated on selective media at 30 °C for 5 days. Next, individual colonies were picked and transferred to fresh DPY-KCl plates for sporulation and isolation of genetically pure clones. For each strain, 3 individual clones were selected and used for the following experiments.

### 4.5. Cultivation and Extraction of Fungal Metabolites

The fungal strains were inoculated with cotton sticks from spore suspensions onto MPY agar (30 g/L maltose; 10 g/L peptone; 5 g/L yeast extract; 0.5 g/L MgSO_4_·7H_2_O; 5 g/L KH_2_PO_4_; microagar 15 g/L in ddH_2_O; pH 5.5), where maltose acts as an inducer for the amyB promoter [60]. The plates were incubated at 30 °C for 5 days prior to extraction. For extraction of intra- and extracellular metabolites, the whole agar pads (medium and mycelium) were sliced into ~1 cm^3^ cubes and transferred to 50 mL polypropylene tubes, spiked with 10 µL of caffeine standard solution (10 mg/mL), then extracted once with 25 mL of 9:1 ethyl acetate–methanol (*v*/*v*) supplemented with 0.1% formic acid. Extraction was performed in a sonication bath for 1 h. The extracts were collected in clean glass vials and dried under a gentle stream of N_2_ at room temperature. The residues were resuspended in 1 mL of 1:1 methanol-ultrapure water (*v*/*v*) supplemented with 0.1% formic acid by pipetting and vortexing, filtered with 0.45 μm PTFE filters into clean HPLC glass vials, and stored at −20 °C until analysis.

### 4.6. HPLC-DAD-MS and HRMS-MS Analysis

HPLC-DAD-MS analysis of fungal extracts was performed using a Waters Acquity Arc HPLC system coupled to a 2998 PDA detector and a QDa single-quadrupole mass spectrometer (Waters, Milford, MA, USA). A Waters XBridge BEH C18 reversed-phase column was applied for separation (50 mm × 2.1 mm I.D., 3.5 μm, 130 Å particles), maintained at 40 °C. The mobile phase consisted of a gradient of solution A (0.1% formic acid in ultrapure water) and solution B (0.1% formic acid in acetonitrile). A split gradient was used: 0–2 min 5% B, 2–10 min linear increase to 50% B, 10–15 min linear increase to 90% B, 15–17 min held at 90% B, 17–17.01 min decrease to 5% B, and 17.01–20 min held at 5% B. The injection volume was 2 µL, and the flow rate was set to 0.5 mL/min. MS analysis was performed in negative and positive ionization modes (ESI), with the following parameters: probe temperature of 600 °C; capillary voltage of ±1.0 kV; cone voltage of ±15 V; scan range 100–1250 *m/z*. For diode array detection (DAD), the wavelength range was set at 190–800 nm. MassLynx v4.2 was used to analyze data obtained in these experiments.

High-resolution tandem mass spectrometry (HRMS-MS) analysis of fungal extracts was performed using a Shimadzu Nexera X2 high-performance liquid chromatography (HPLC) system with a binary LC20ADXR pump coupled to a Thermo Scientific Q Exactive plus hybrid quadrupole-orbitrap mass spectrometer (Thermo Fisher Scientific, Waltham, MA, USA). A Kinetex EVO C18 reversed-phase column was applied for HPLC separations (100 mm ×  2.1 mm I.D., 2.6 μm, 100 Å particles) (Phenomenex, Torrance, CA, USA), maintained at 40 °C. The mobile phase consisted of a gradient of solution A (0.1% formic acid in ultrapure water) and solution B (0.1% formic acid in acetonitrile). A linear gradient was used: 0–2 min 5% B, 2–21 min linear increase to 50% B, 21–27.5 min linear increase to 90% B, 27.5–30 min held at 90% B, 30–30.5 min decrease to 5% B, and 30.5–40 min held at 5% B. The injection volume was 2 µL, and the flow rate was set to 0.4 mL/min. MS and MS/MS analyses were performed with heated electrospray ionization (HESI) in positive mode. The spray voltage was set to 3.5 kV, and the sheath and auxiliary gas flow were set at 47.5 and 11.25, respectively. The ion transfer tube temperature was 256.25 °C. Spectra were acquired in data-dependent mode with a survey scan at *m/z* 100–1500 at a resolution of 70,000, followed by MS/MS fragmentation of the top 5 precursor ions at a resolution of 17,500. A normalized collision energy of 30 was used for fragmentation, and fragmented precursor ions were dynamically excluded for 4 s. MZmine 3 [61] was used to analyze data obtained in these experiments.

## Figures and Tables

**Table 1:** Predicted function of genes from the putative hericenone BG. For *herA*, *herB*, and g074890, amino acid sequence similarity and identity to the corresponding homologous proteins from meroterpenoid-producing *stb* BGC (MIBiG no. BGC0001390) [34] and *asc* BGCs (MIBiG no. BGC0001923 and BGC0001924) [35] are shown.

Gene	Predicted Function	*stb* BGC Homolog (aa % Identity; % Similarity)	*asc* BGC Homolog (aa % Identity; % Similarity)
g019500	Flavoprotein		
g019520	Fungal-specific transcription factor		
g019530	NRPS-like reductase		
g019540	Aldehyde dehydrogenase		
**g019550_*herA***	**Type I PKS**	BAV19379.1_StbA (24.89; 41.98)	BBF25315.1_AscC (25.02; 41.92)
g019560	MFS 1 family transporter		
g019580	short-chain dehydrogenase/reductase SDR		
g019590	Monooxygenase FAD-binding		
**g019600_*herB***	**Carboxylic acid reductase**	BAV19380.1_StbB (31.71; 50.75)	BBF25314.1_AscB(32.10; 50.13)
g019620	Phenylalanine-specific permease		
g019630	Serine/threonine protein kinase		
**g074890**	**Prenyltransferase**	BAV19381.1_StbC (25.79; 44.99)	BBF25313.1_AscA (26.52; 41.44)

## Data Availability

The original contributions presented in this study are included in the main article and additional files. Further inquiries pertaining to fungal strains, plasmids, and raw mass spectrometry data can be directed to the corresponding authors.

## Supplementary Materials

The following supporting information can be downloaded at: https://www.mdpi.com/article/10.3390/molecules29235576/s1, Additional File S1. Supplementary figures and tables (.docx). Additional File S2. BGC predictions in the genome of *H. erinaceus* 0605 by fungiSMASH (sheet 1). Output of pHMMER search of putative prenyltransferase (sheet 2) (.xlsx). Additional File S3. HRMS and MS2 spectra of unidentified compounds from *herAB* strains (.pdf). Additional File S4. Annotation file for *H. erinaceus* 0605 assembly (GeneBank acc. no. GCA_016906435.1) (.zip).
