# Supplementary material for: Identification and Reconstitution of the First Two Enzymatic Steps for the Biosynthesis of Bioactive Meroterpenoids from Hericium erinaceus (Lion’s Mane Mushroom)
Source: Molecules. 2024 Nov 26;29(23):5576. doi: 10.3390/molecules29235576 (PMC11643632; doi:10.3390/molecules29235576)

1. LC-MS *m/z* 259 $[M-H]^-$; HRMS *m/z* 261.1124 $[M+H]^+$

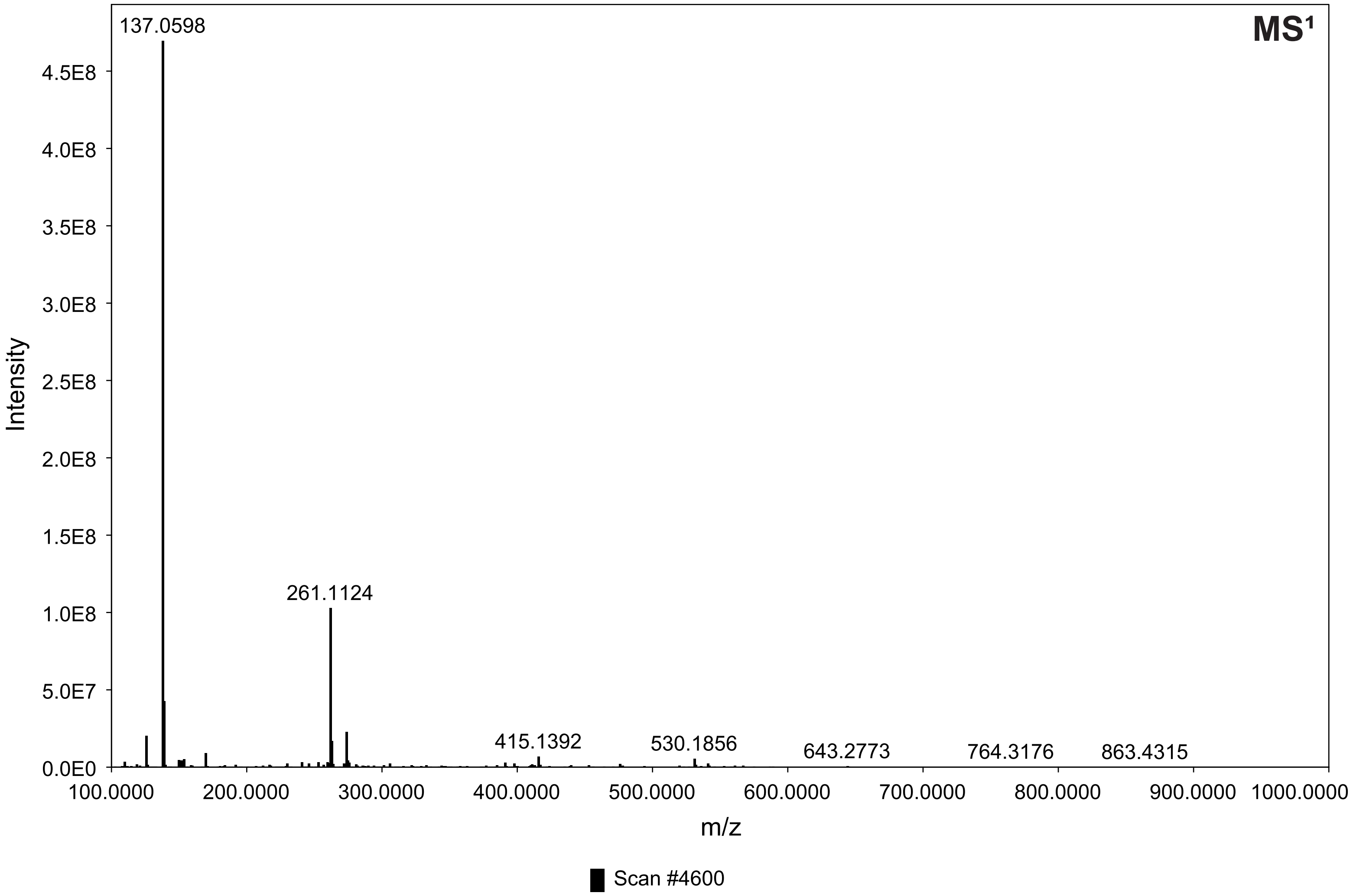

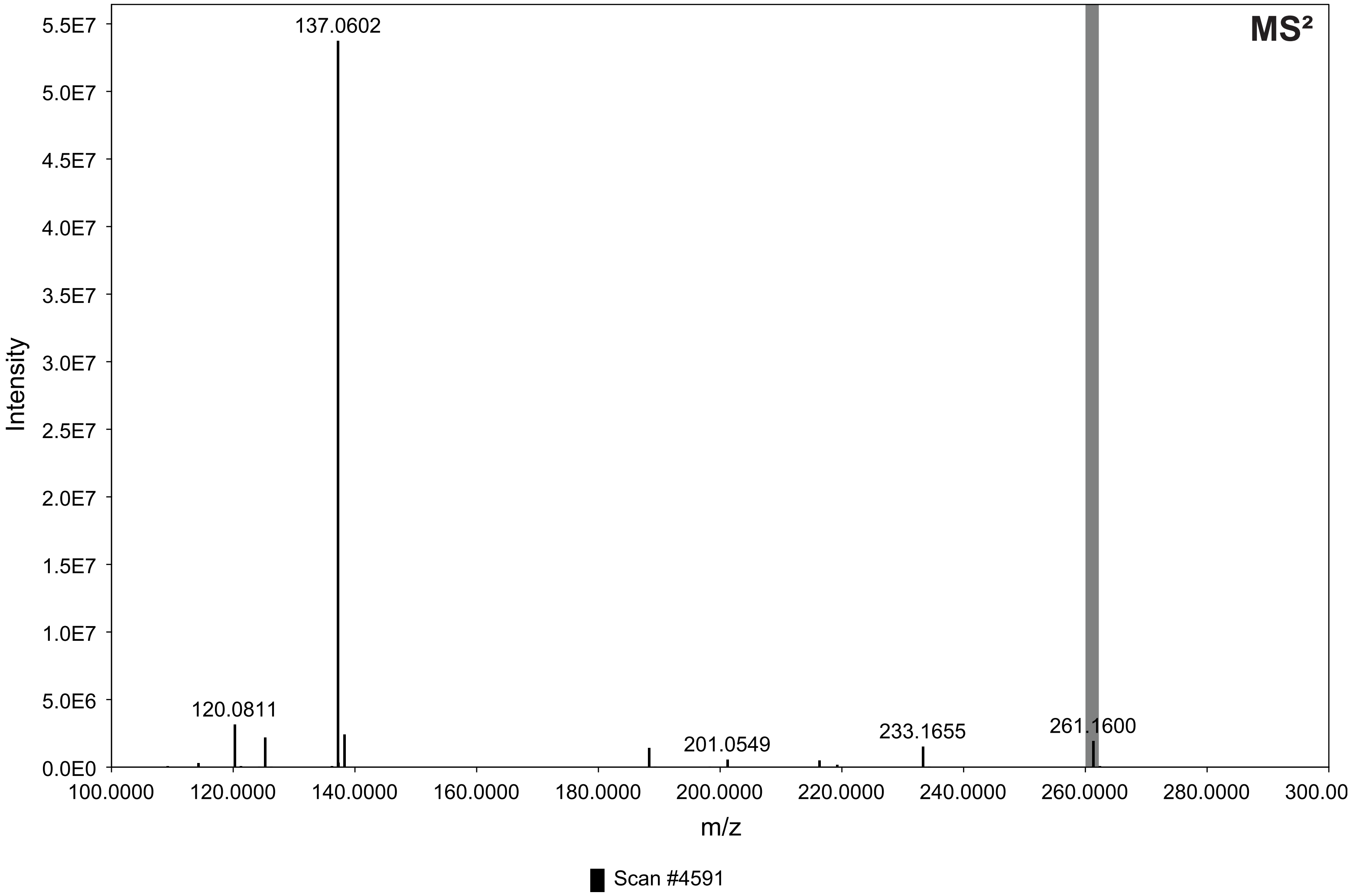

2. LC-MS *m/z* 261 $[M-H]^-$; HRMS *m/z* 263.0915 $[M+H]^+$

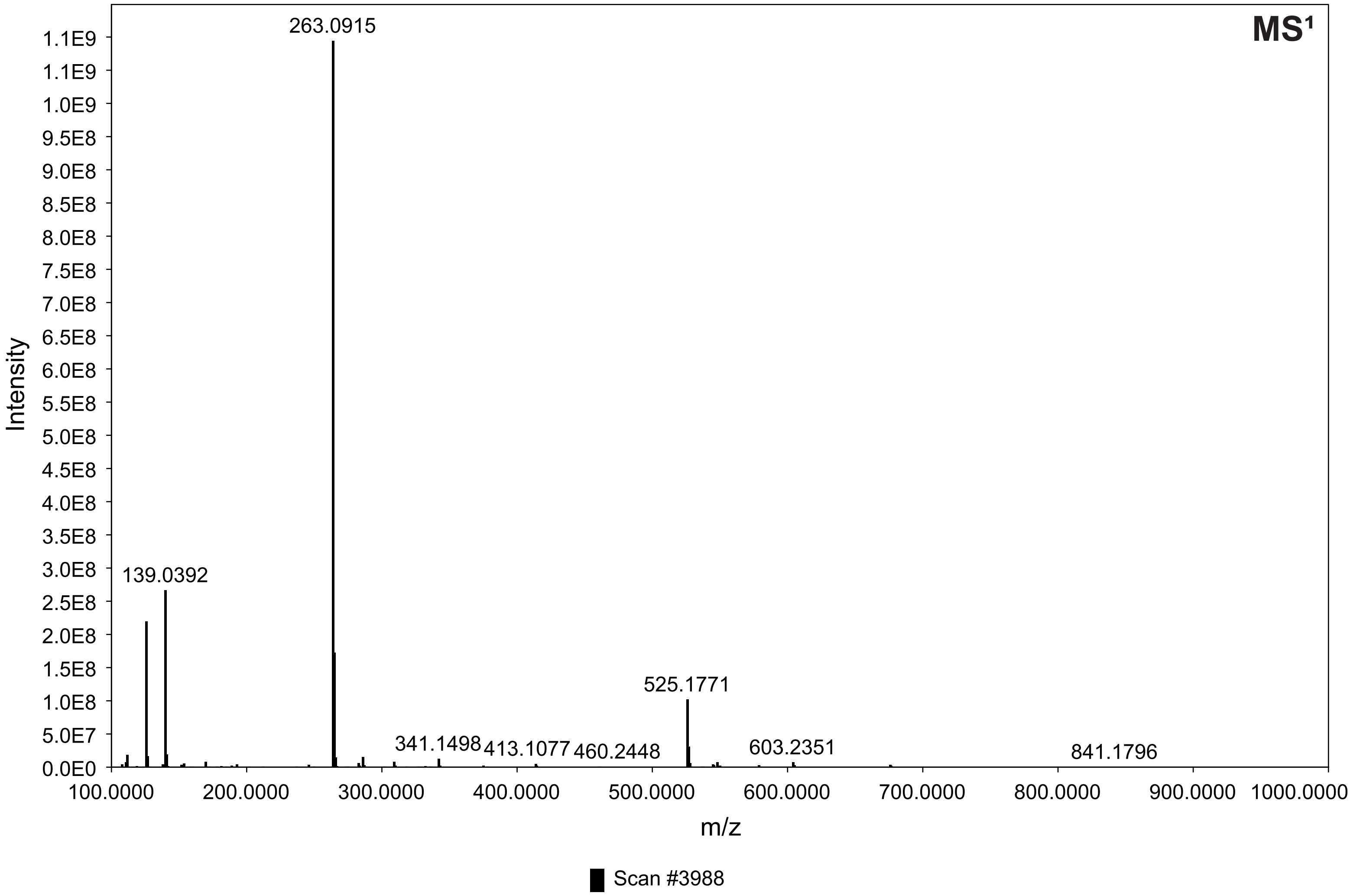

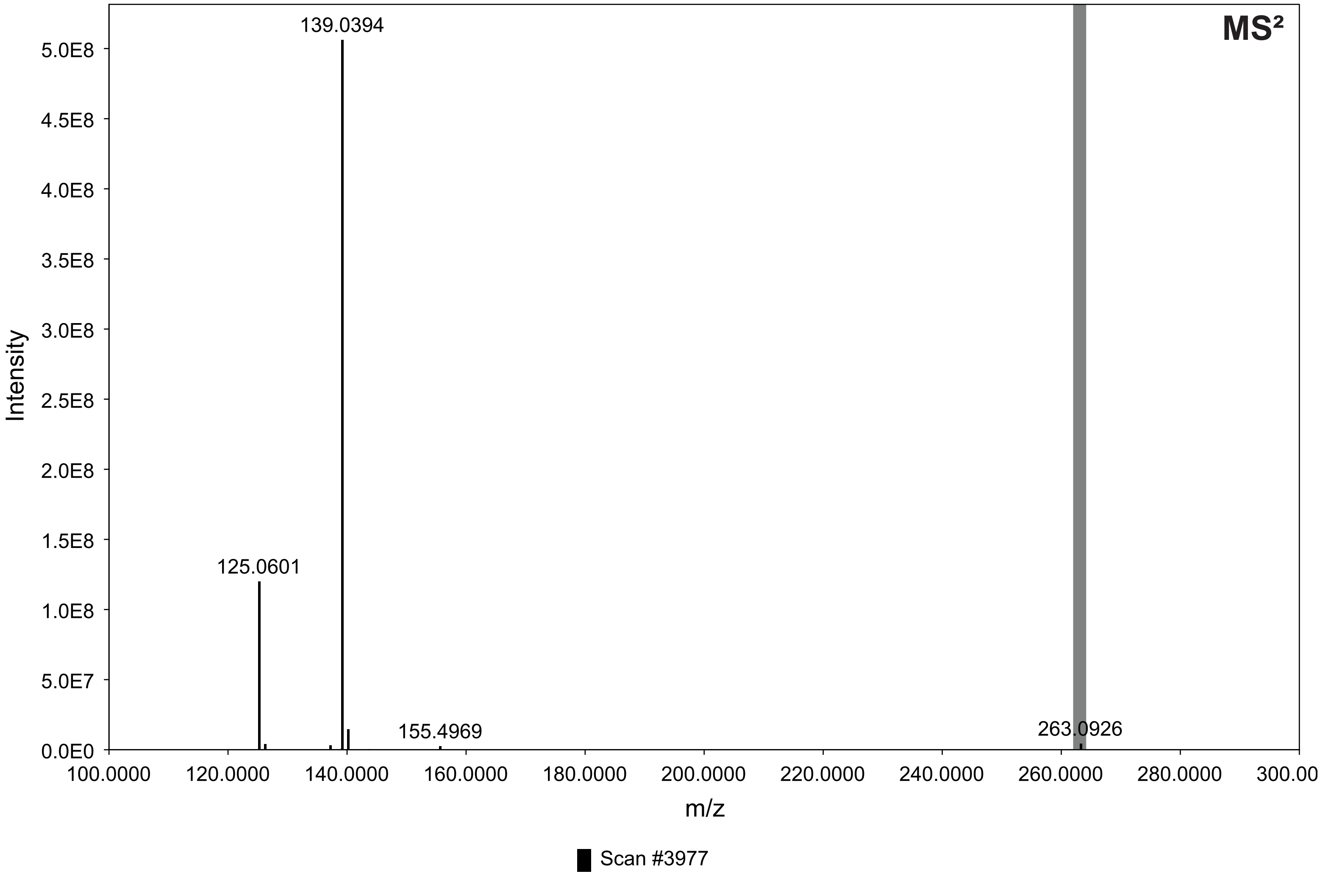

3. LC-MS *m/z* 303 [M-H]$^-$; HRMS *m/z* 305.1021 [M+H]$^+$

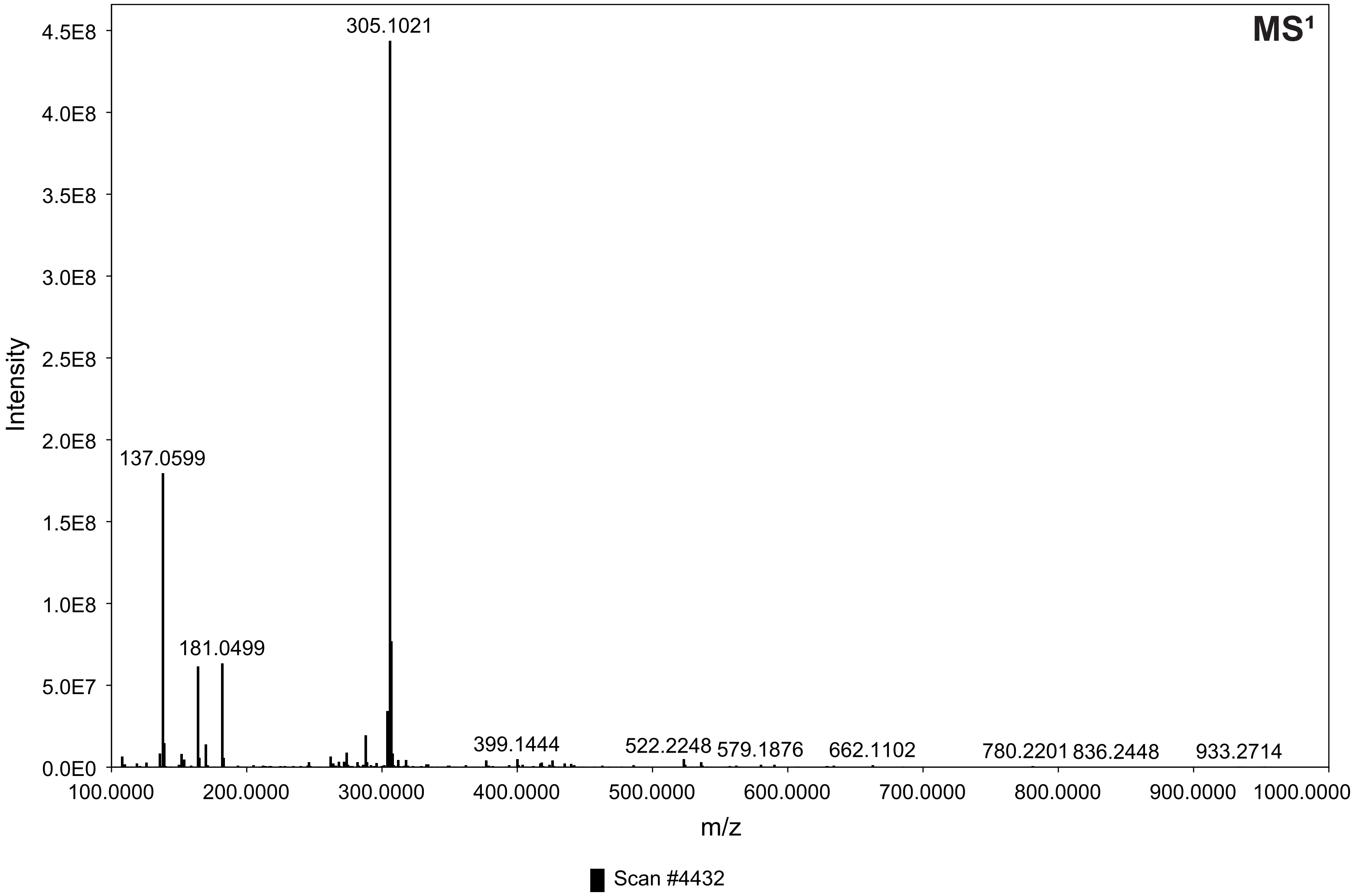

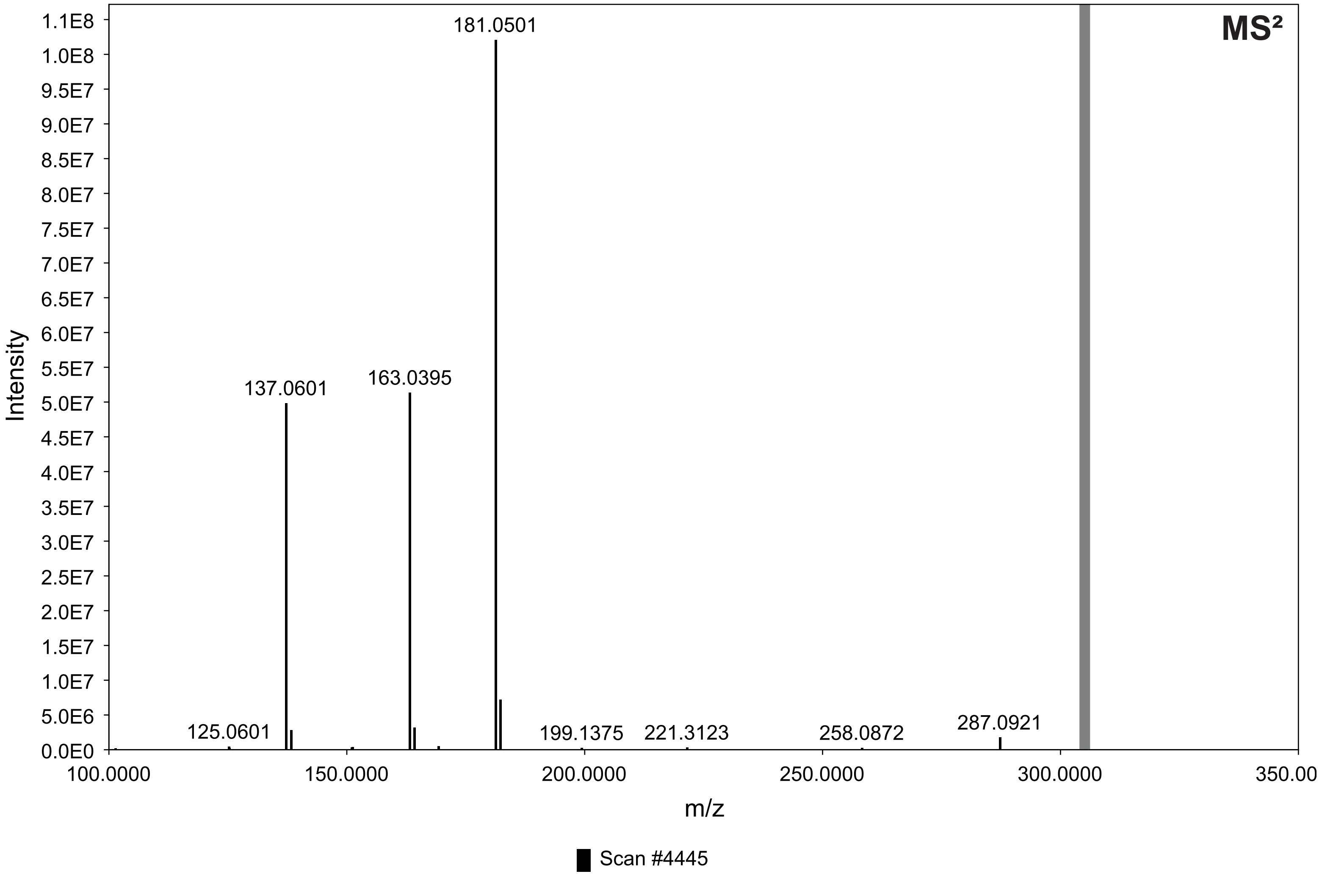

4. LC-MS *m/z* 395 [M-H]$^-$; HRMS *m/z* 397.1648 [M+H]$^+$

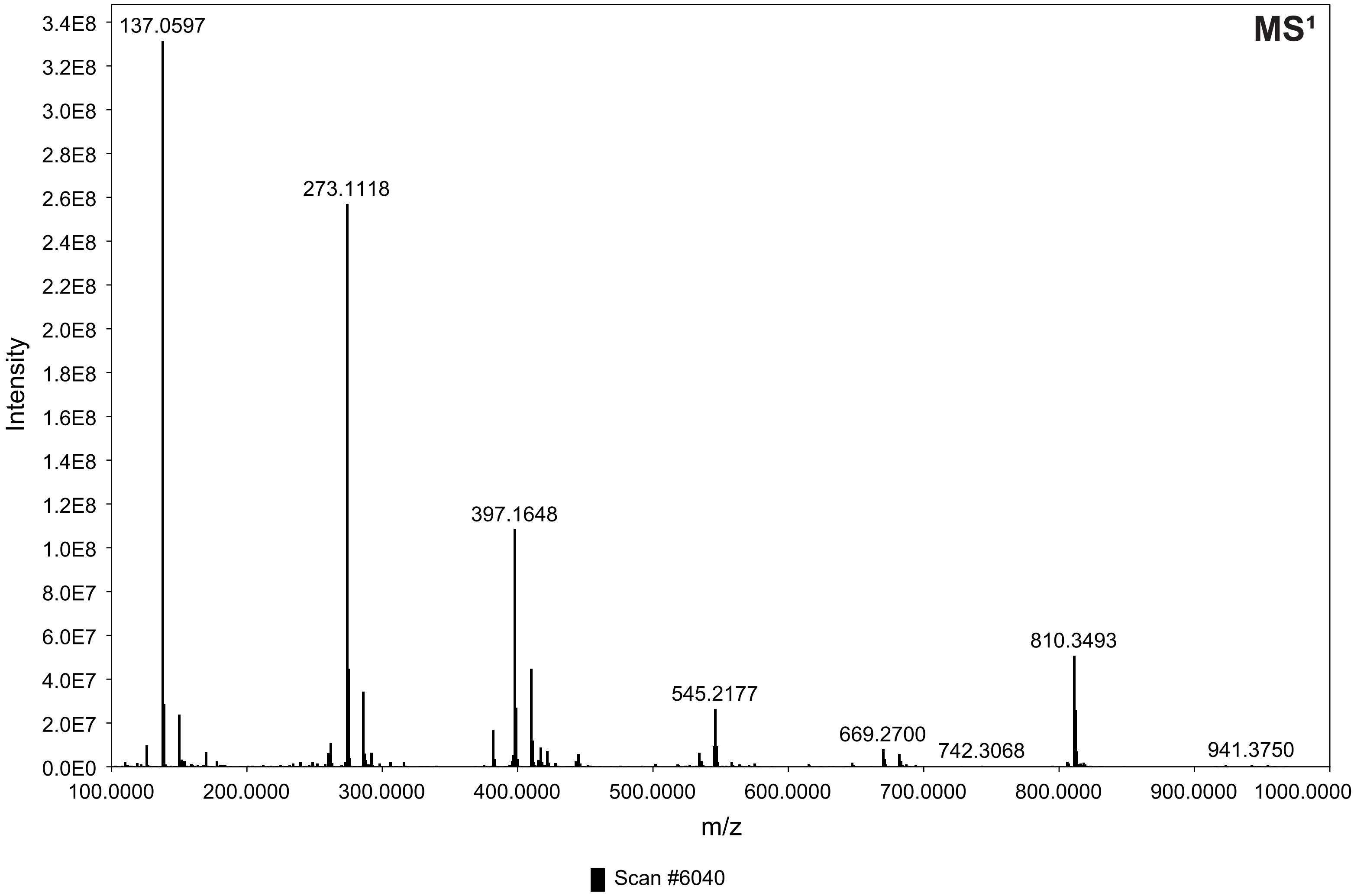

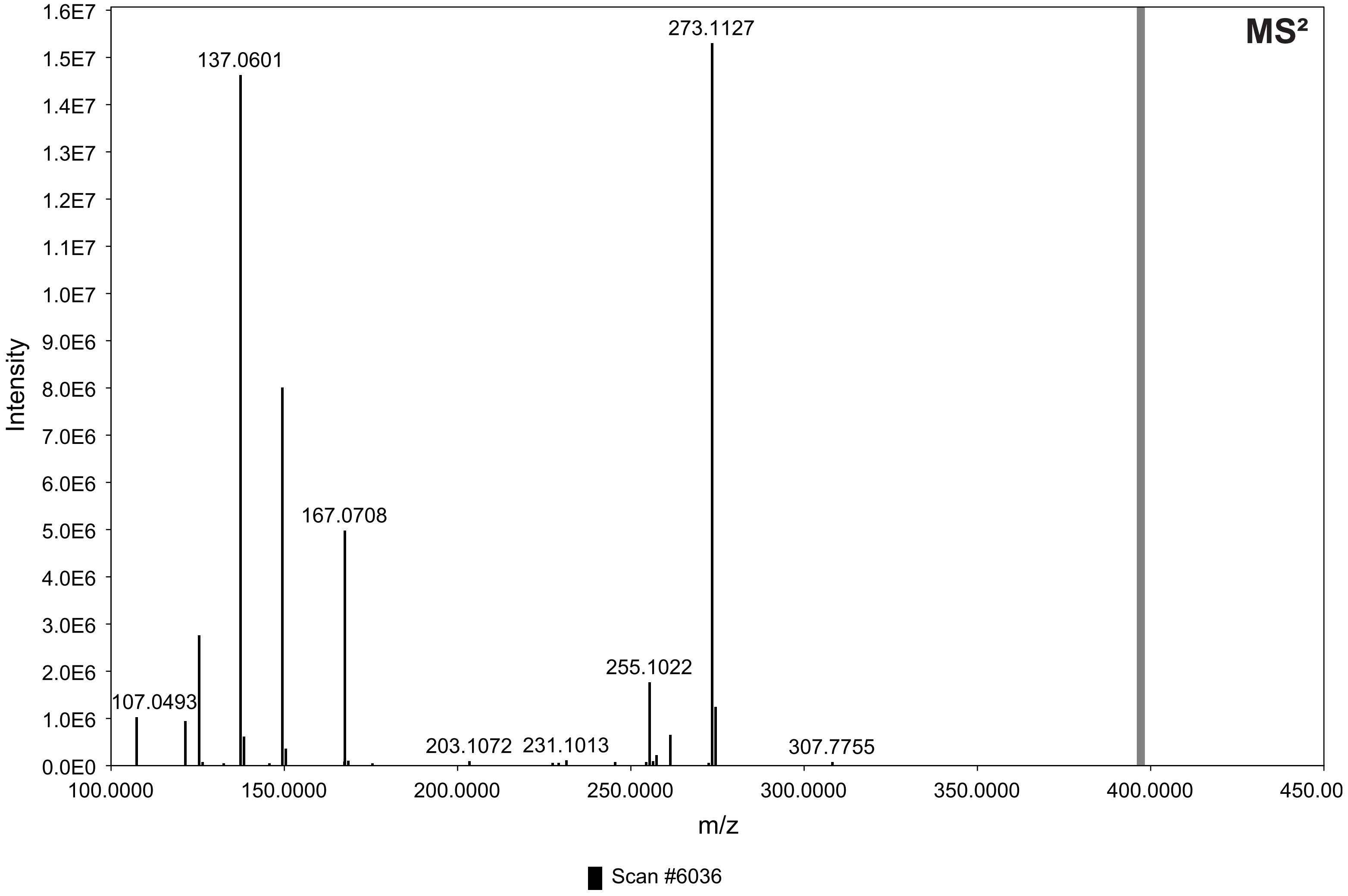

5. LC-MS *m/z* 423 $[M-H]^-$; HRMS *m/z* 425.1599 $[M+H]^+$

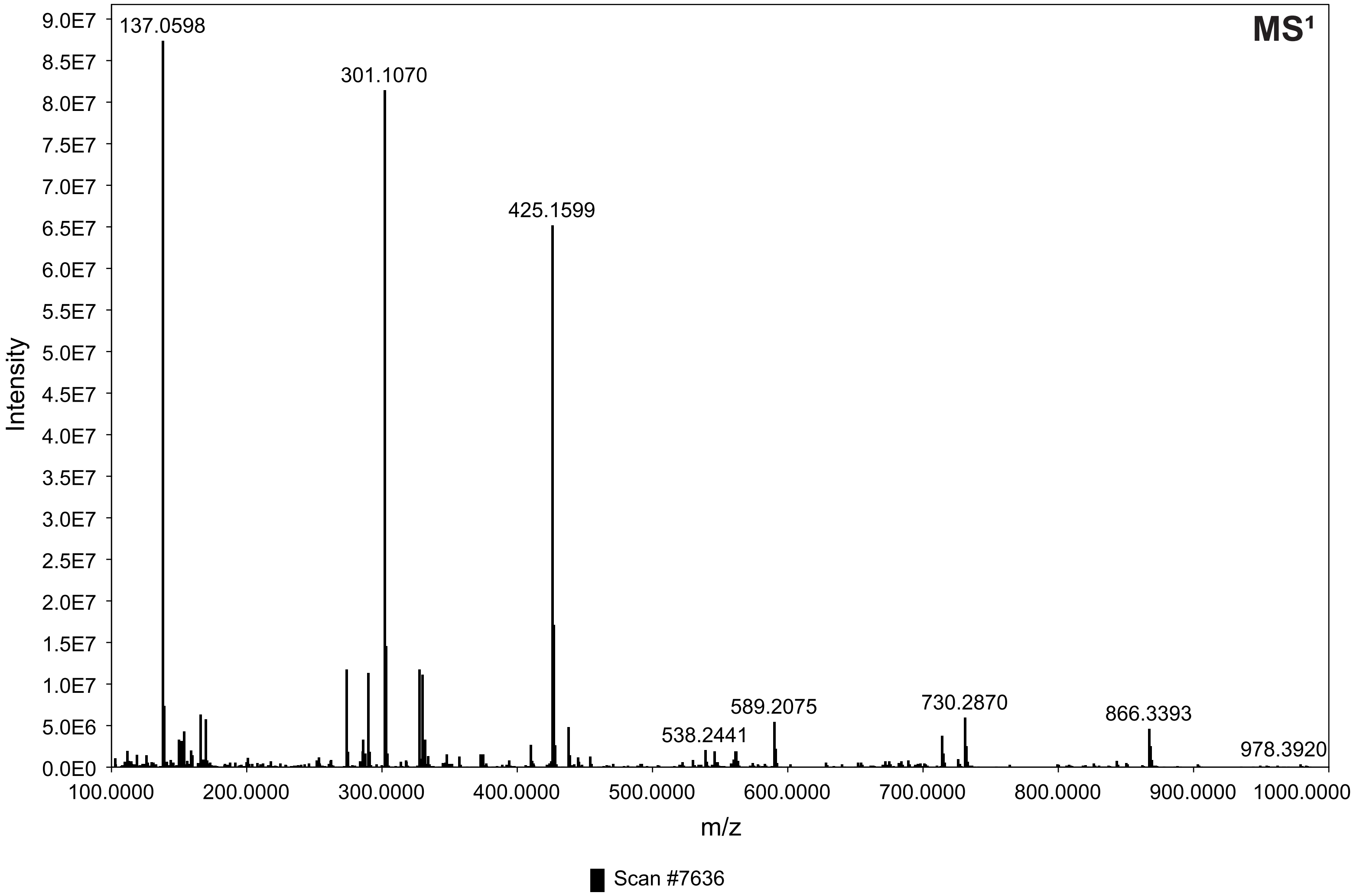

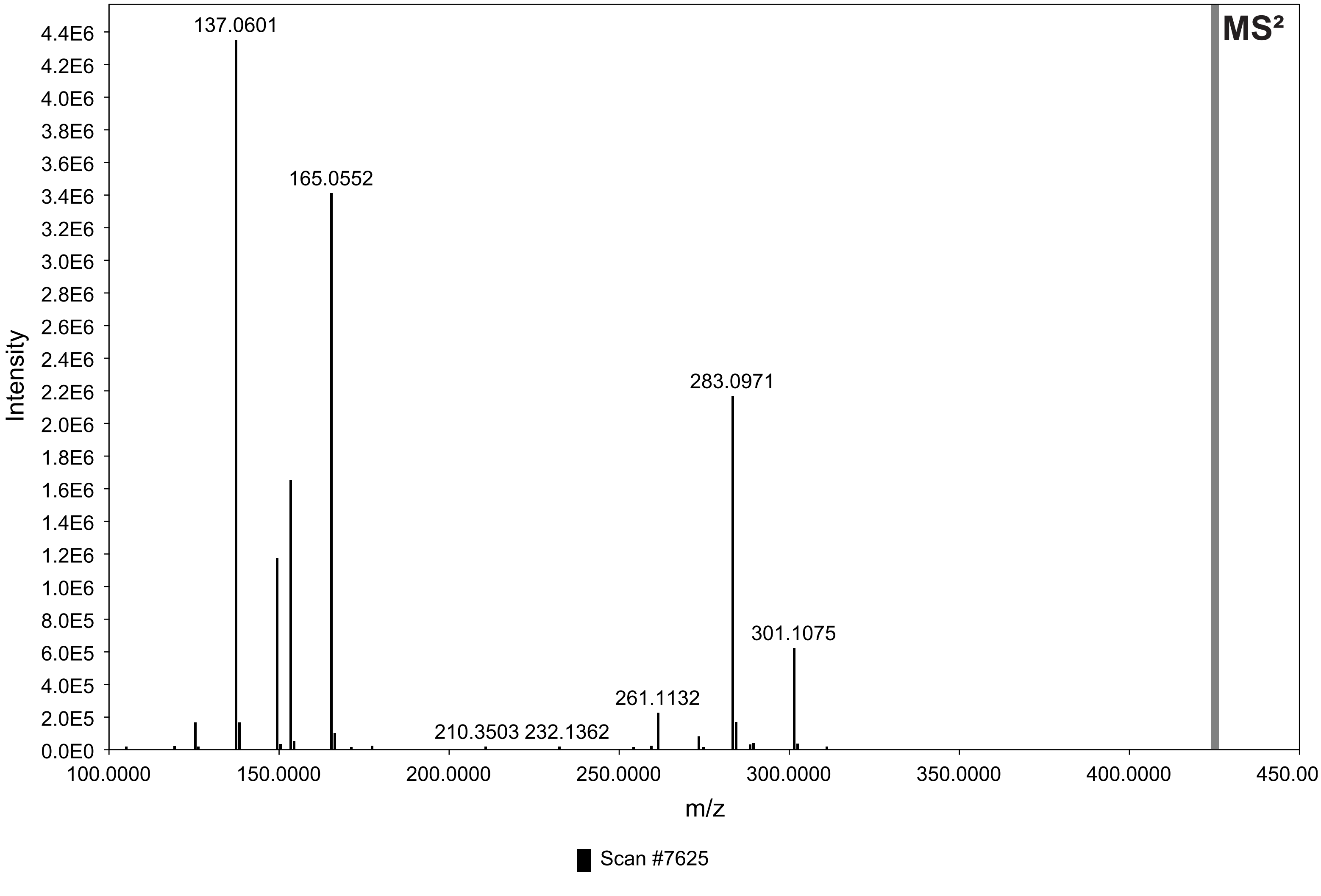

6. LC-MS *m/z* 439 [M-H]⁻; HRMS *m/z* 441.1549 [M+H]⁺

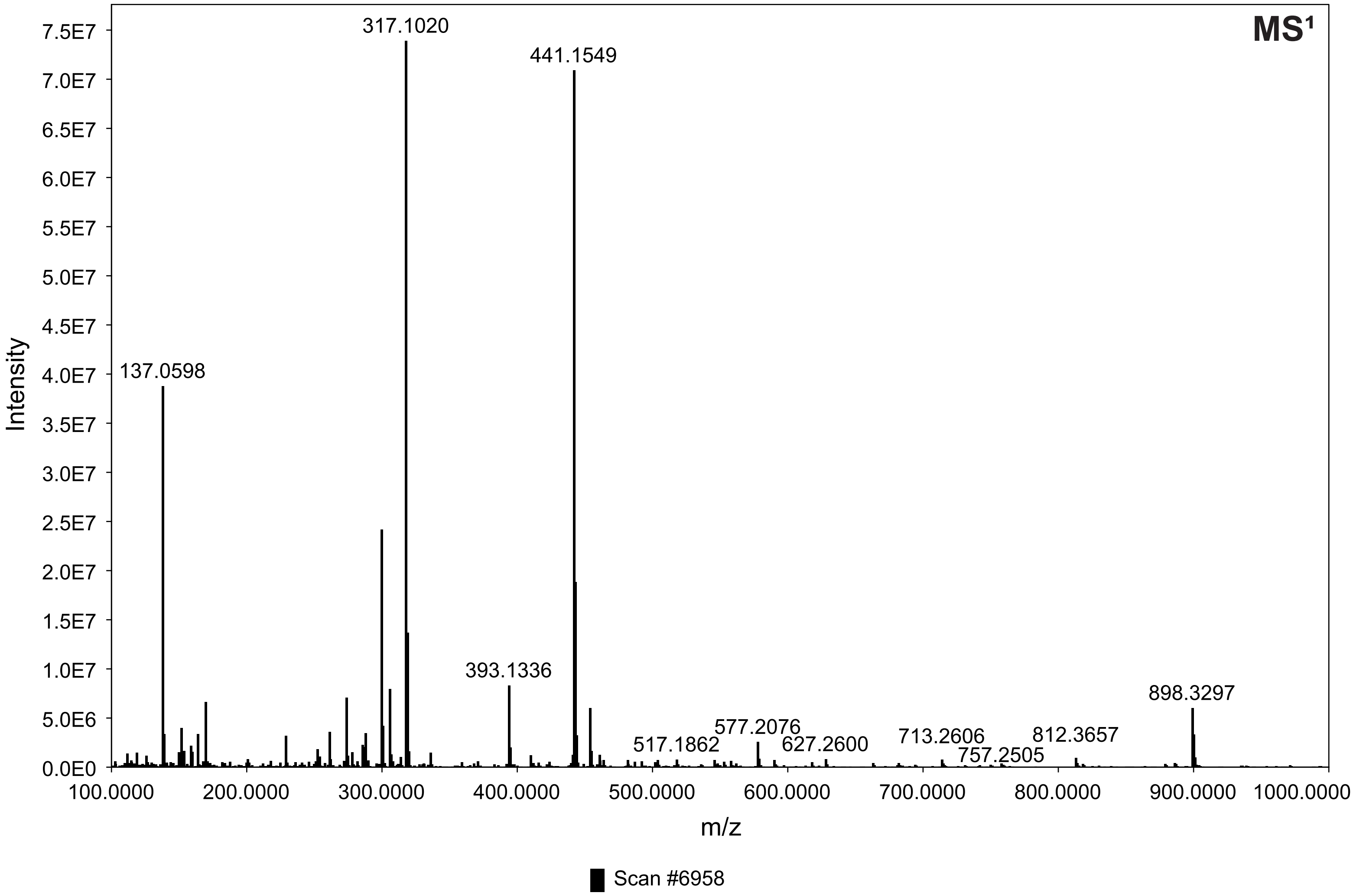

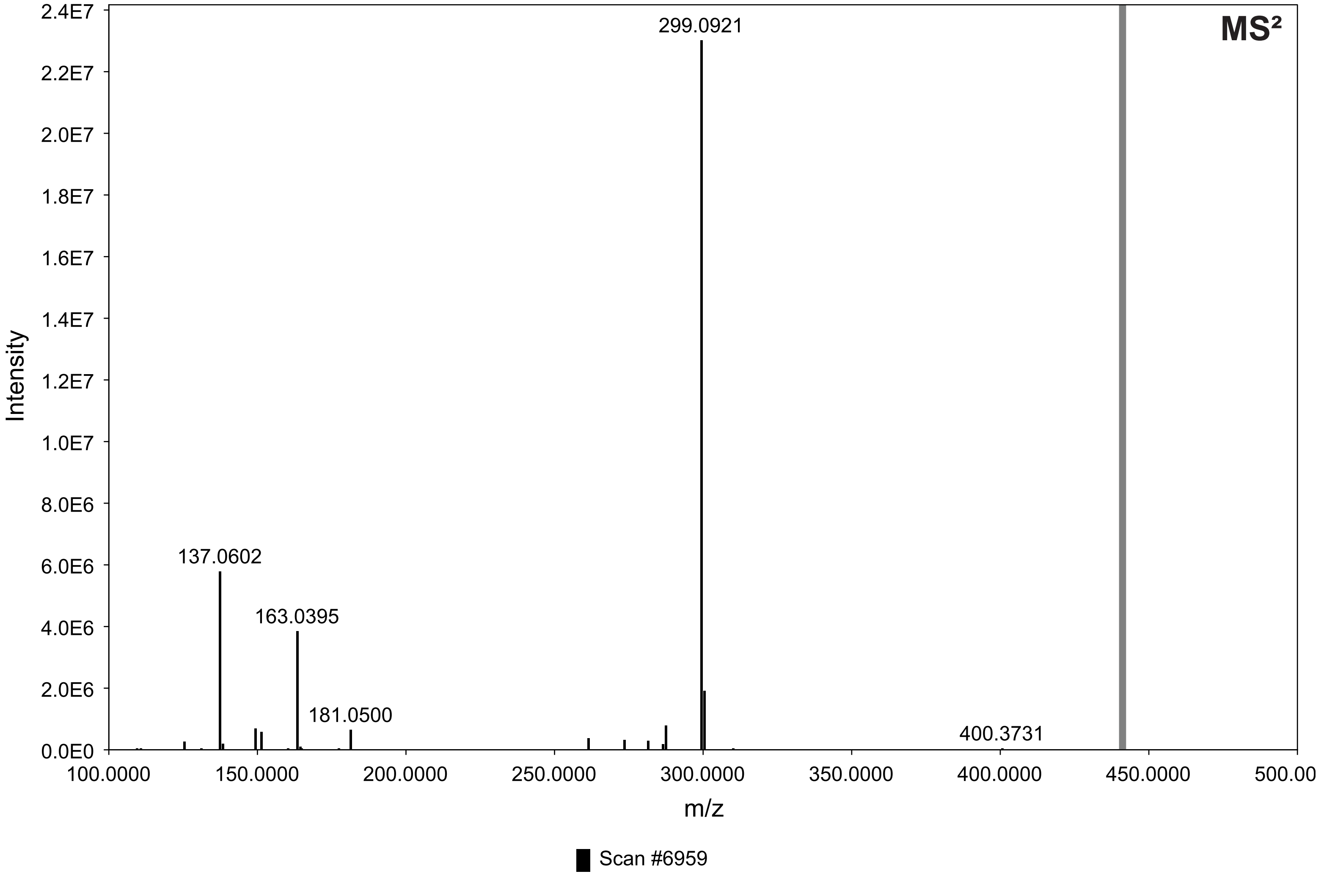

7. LC-MS *m/z* 531 $[M-H]^-$; HRMS *m/z* 533.2176 $[M+H]^+$

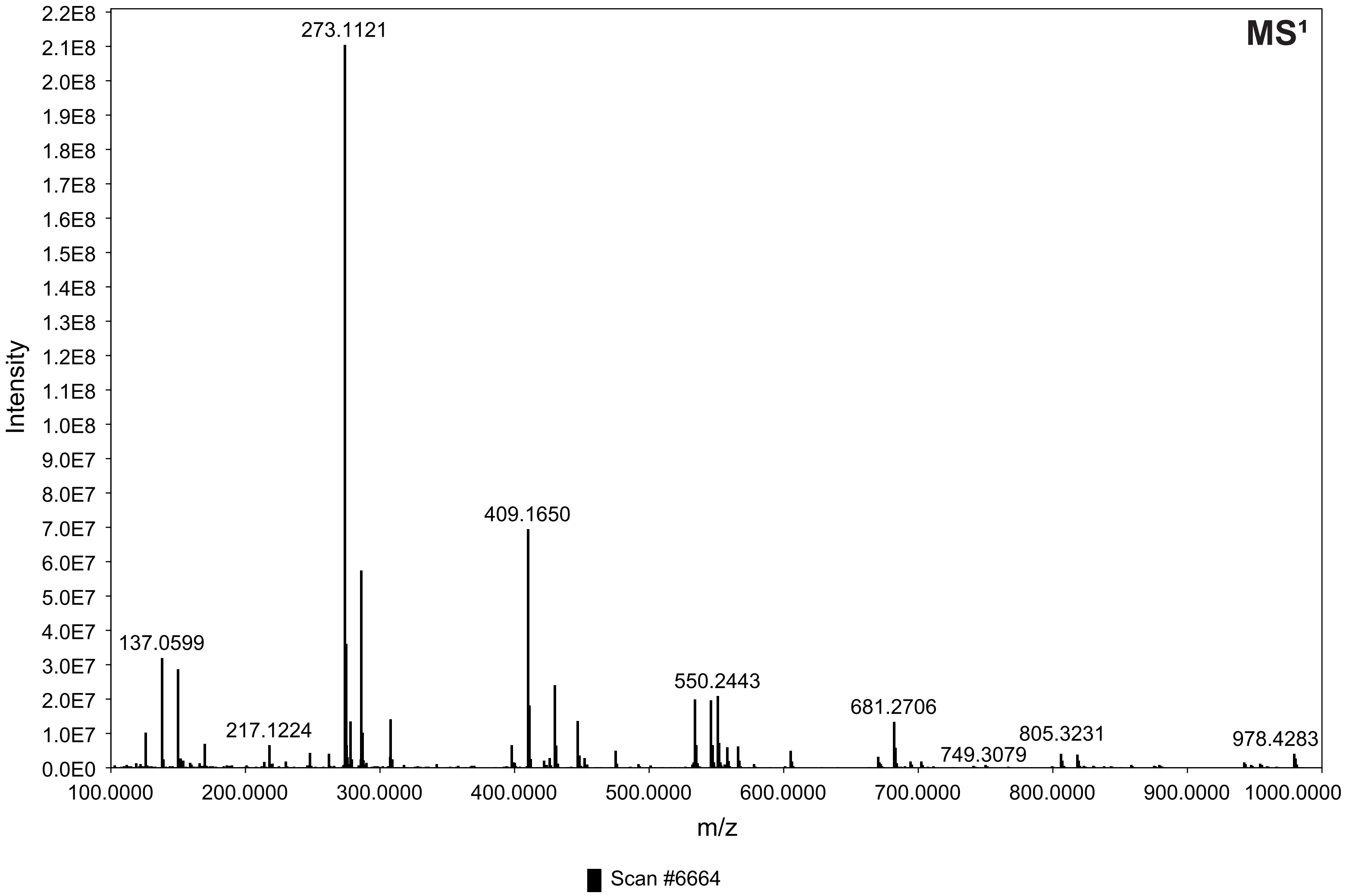

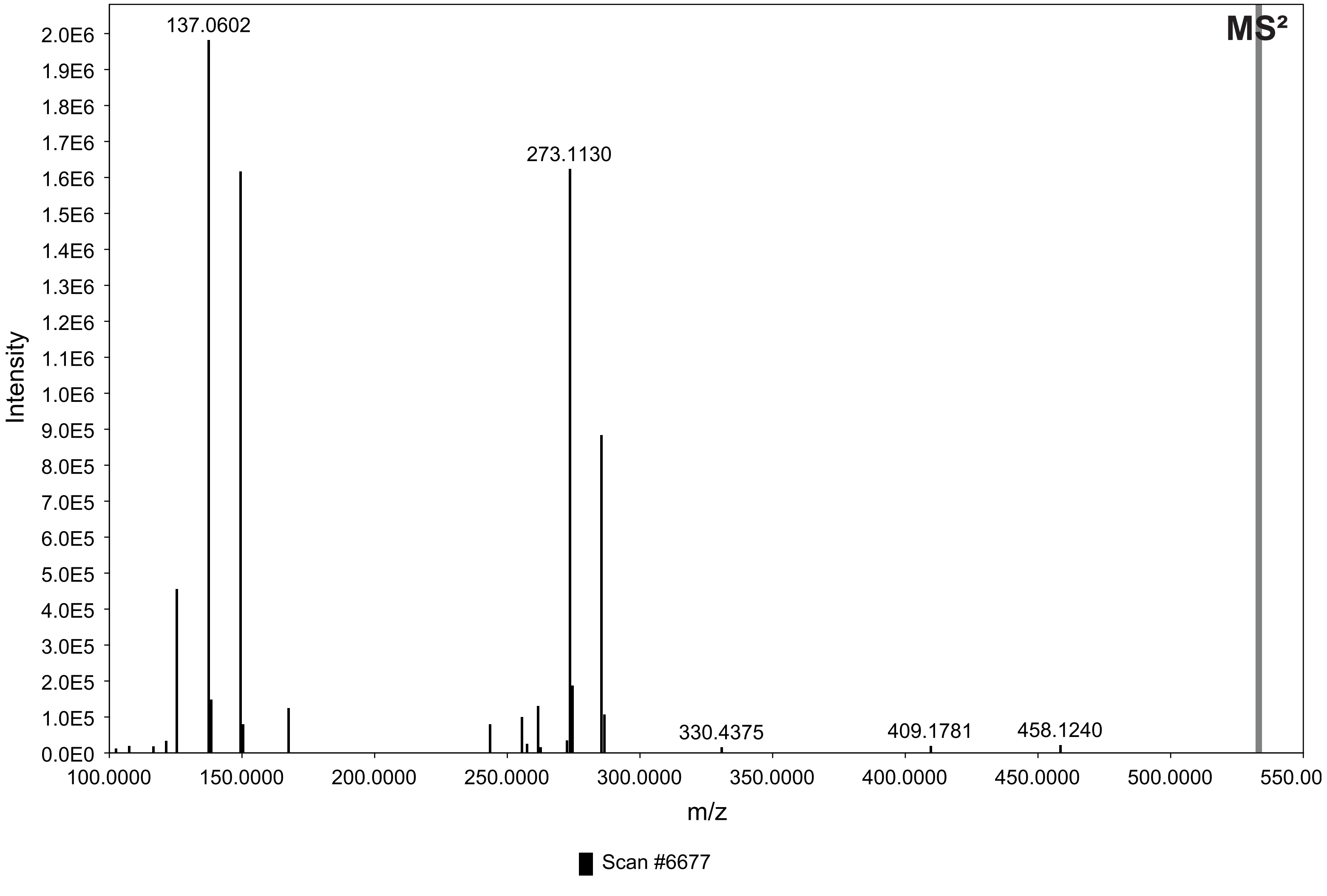

8. LC-MS m/z 575 $[M-H]^-$; HRMS m/z 577.2078 $[M+H]^+$

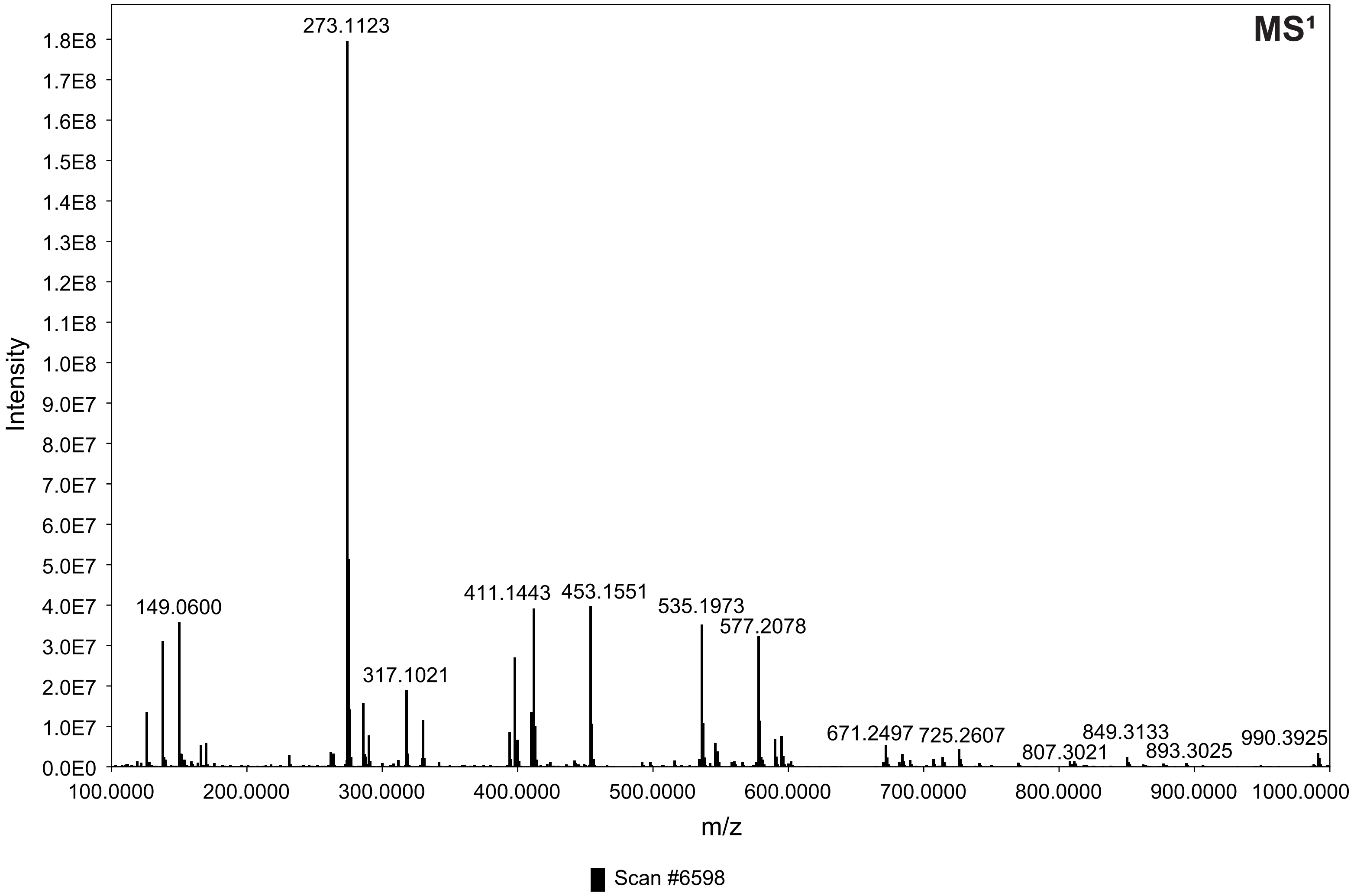

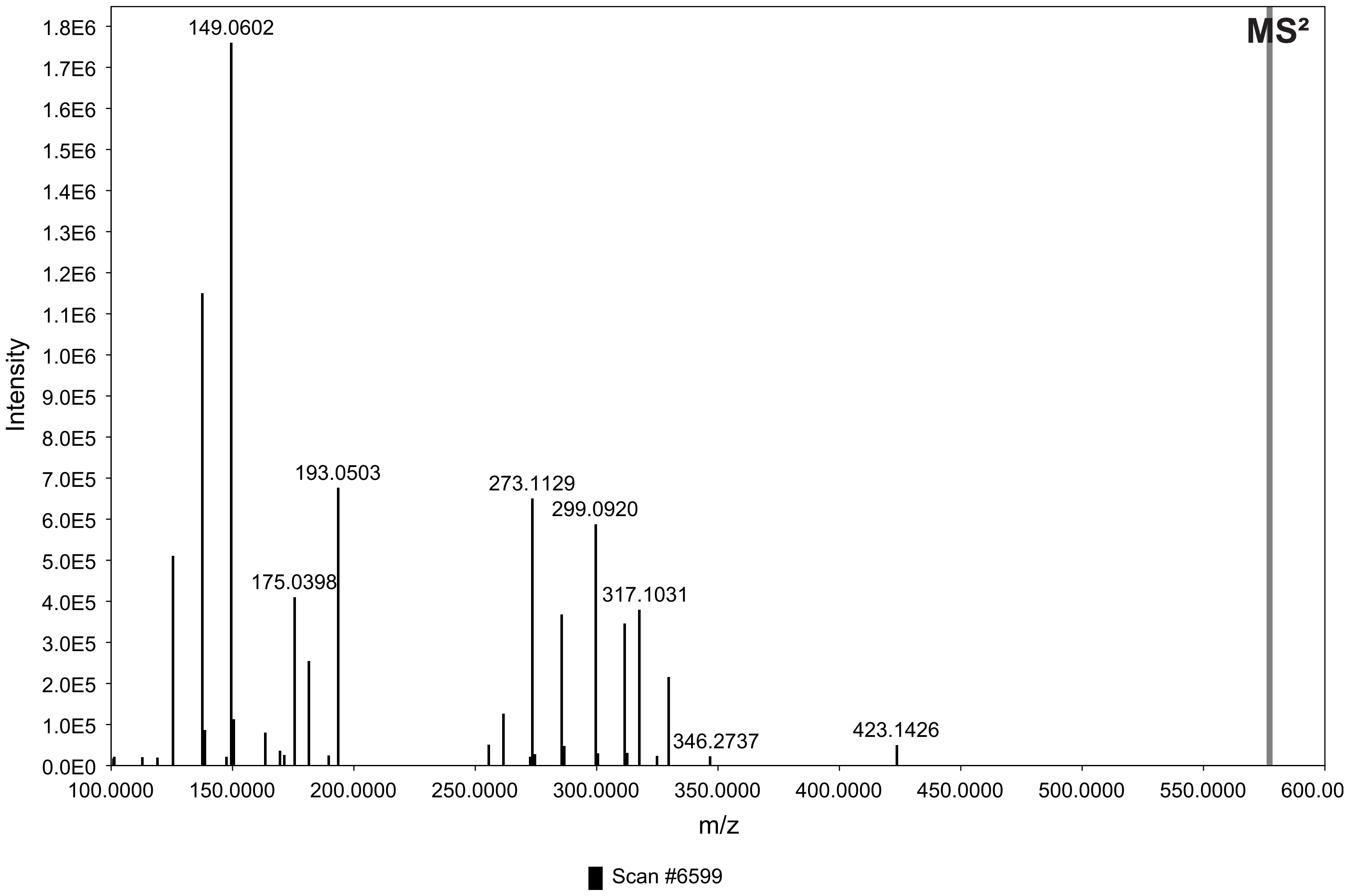

9. LC-MS *m/z* 667 [M-H]⁻; HRMS *m/z* 669.2701 [M+H]⁺

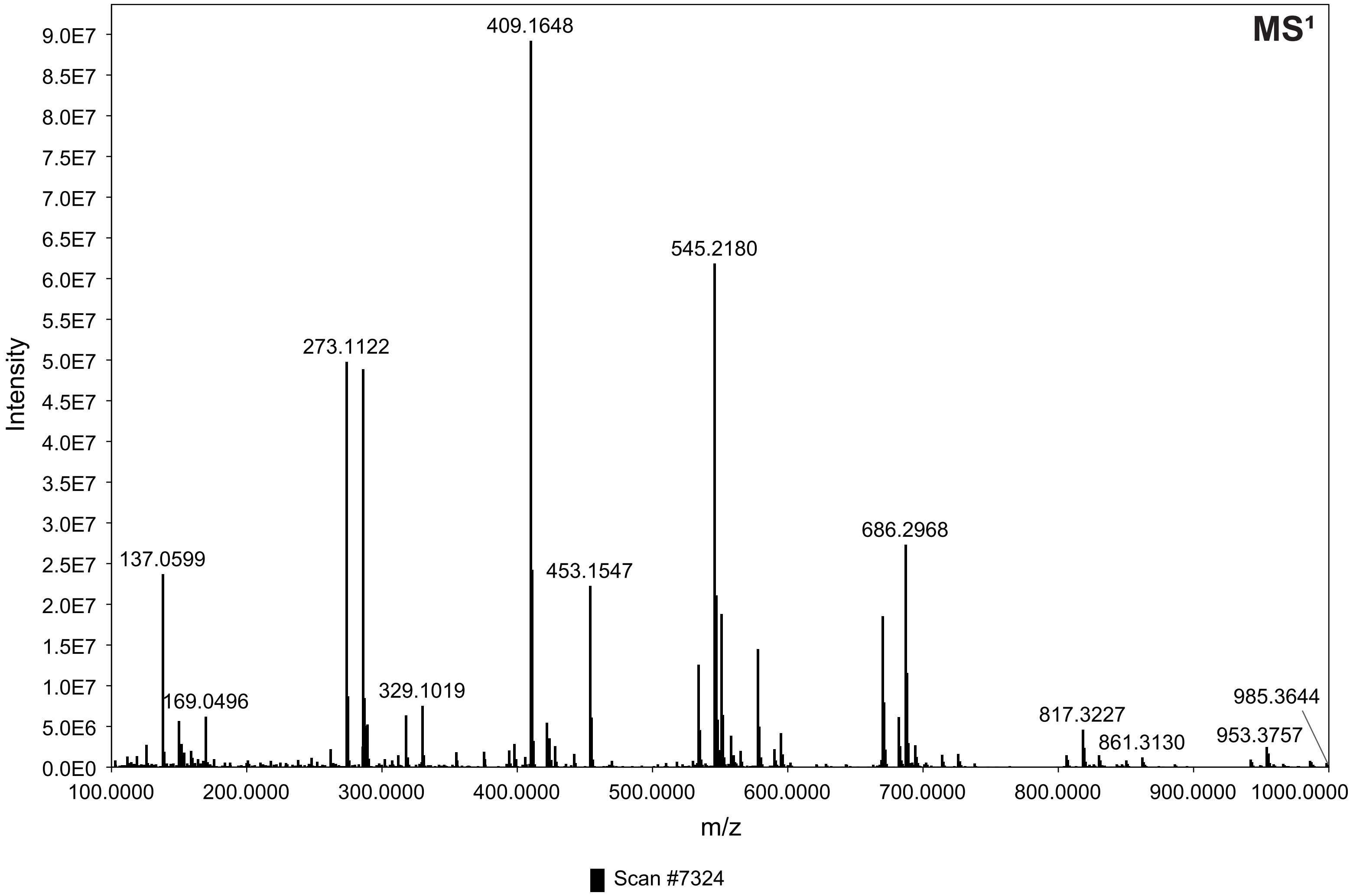

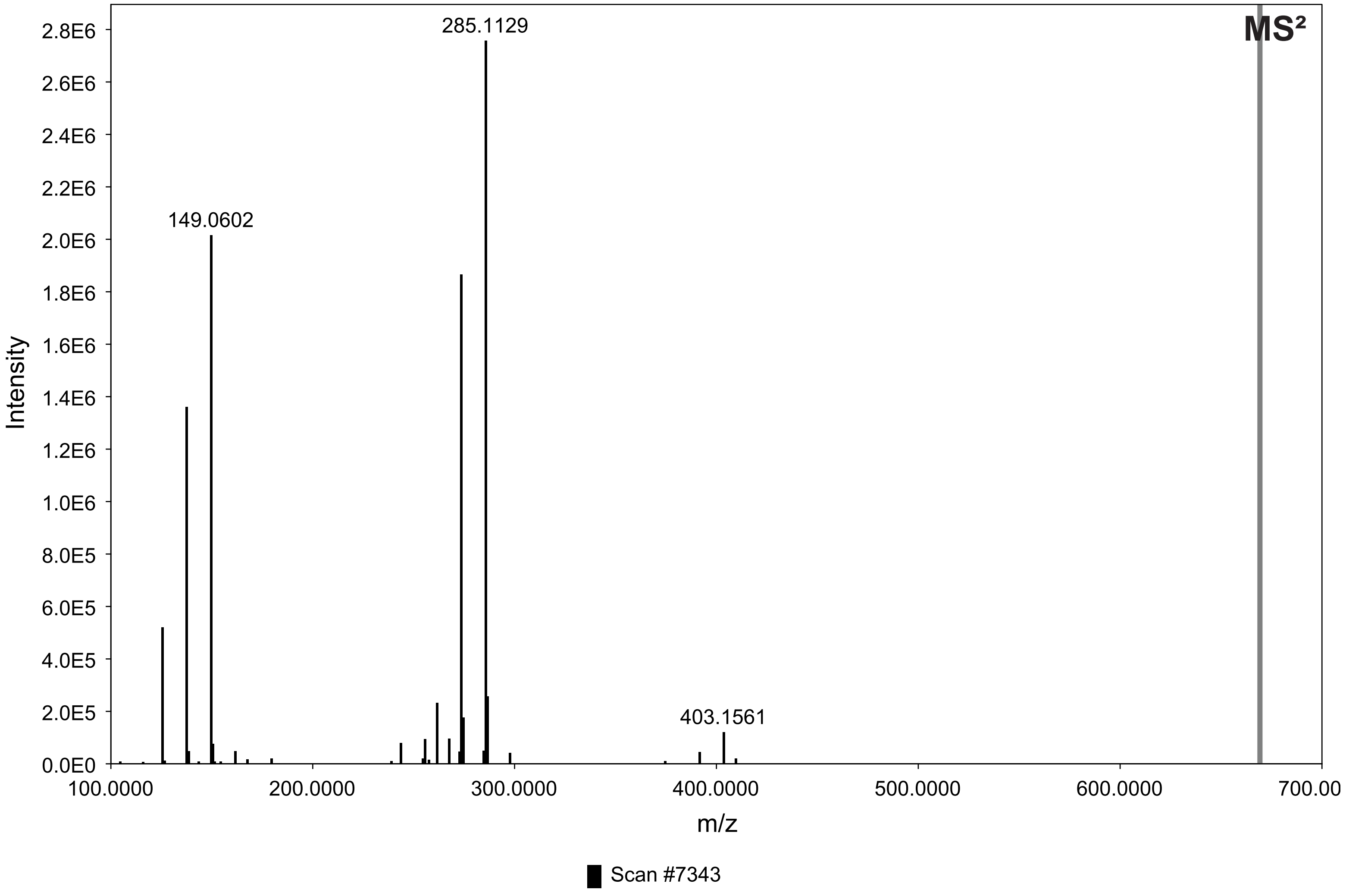

10. LC-MS *m/z* 711 [M-H]$^-$; HRMS *m/z* 713.2601 [M+H]$^+$

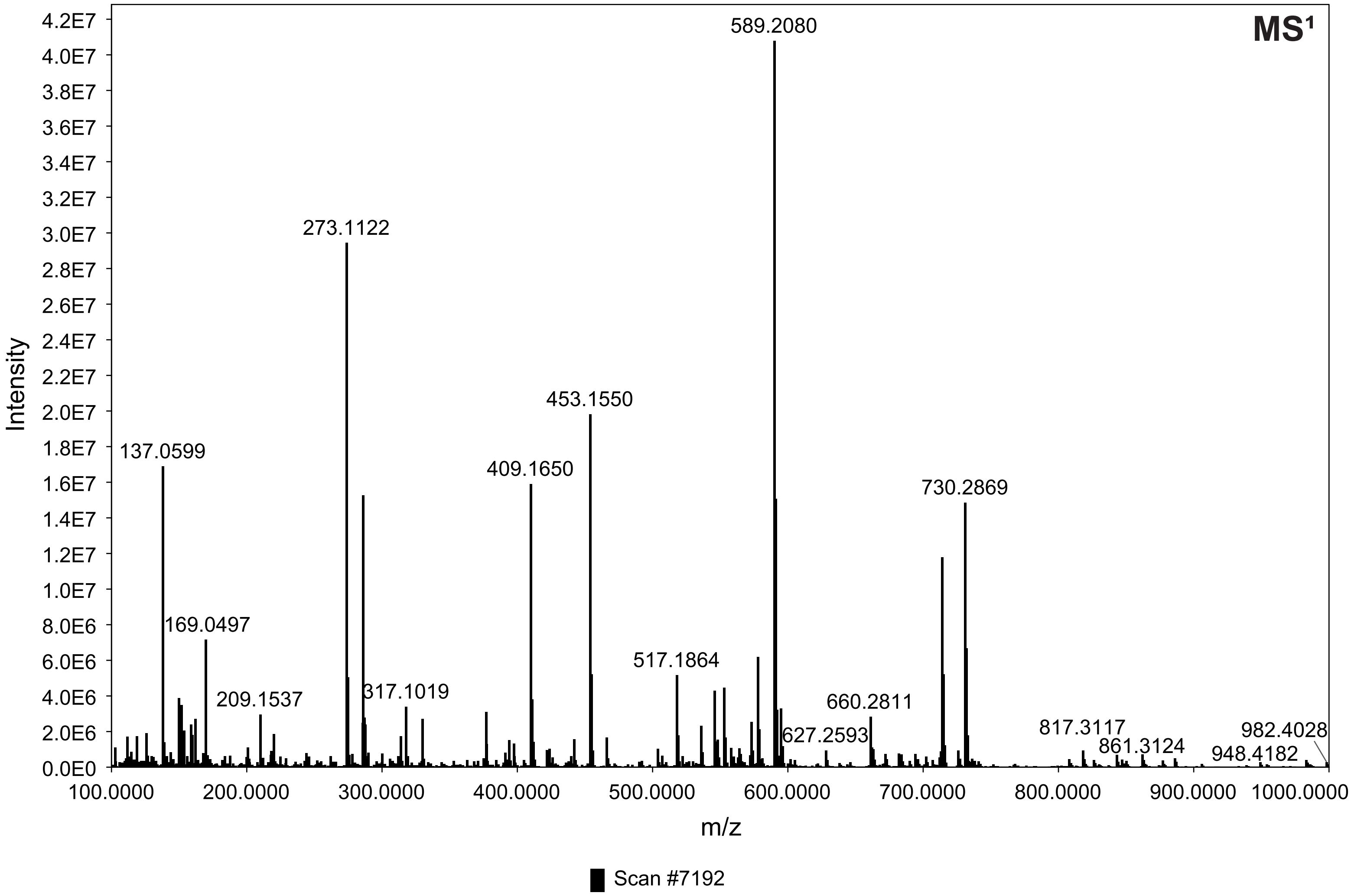

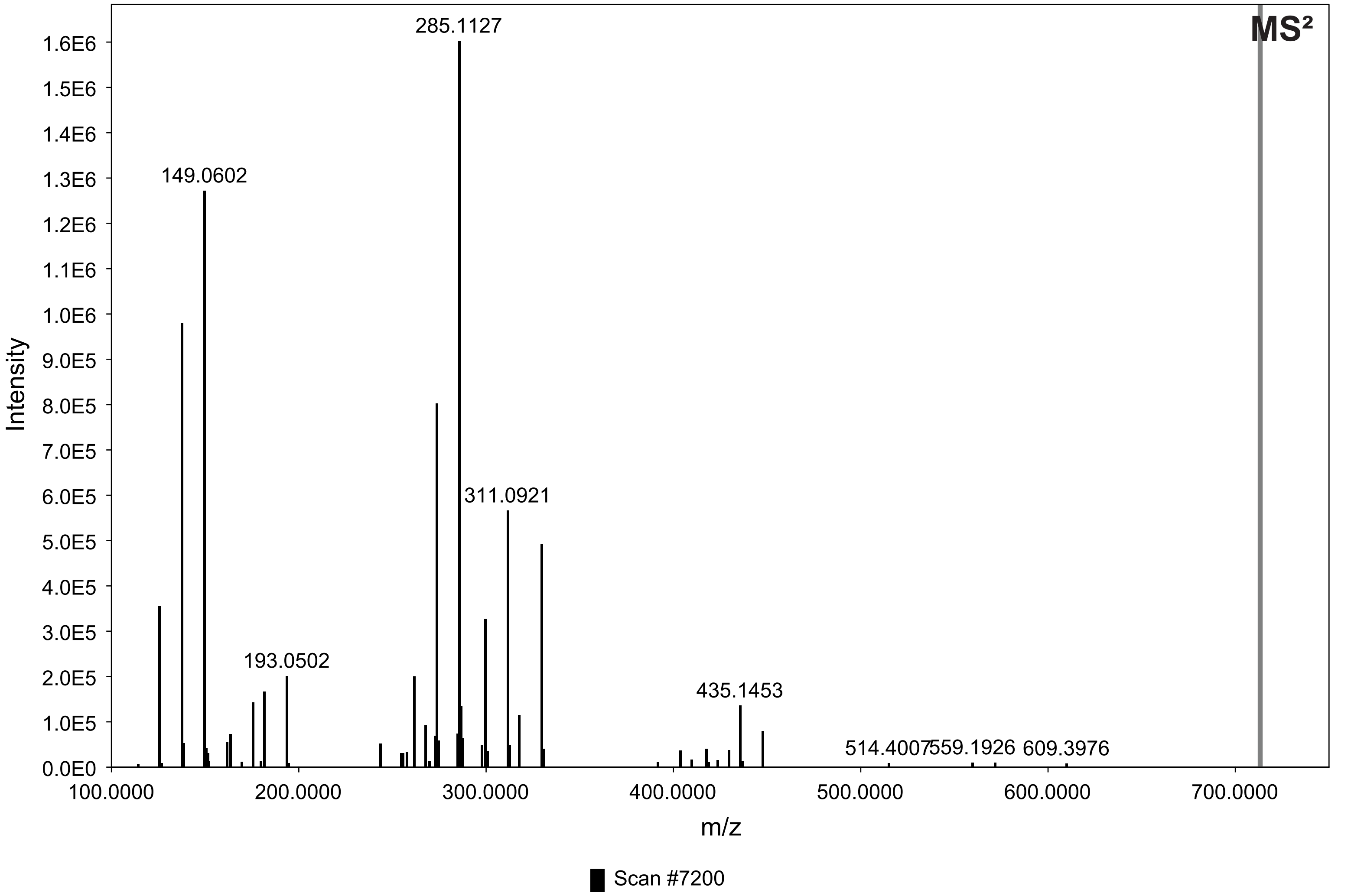

11. LC-MS *m/z* 803 [M-H]$^-$; HRMS *m/z* 805.3232 [M+H]$^+$

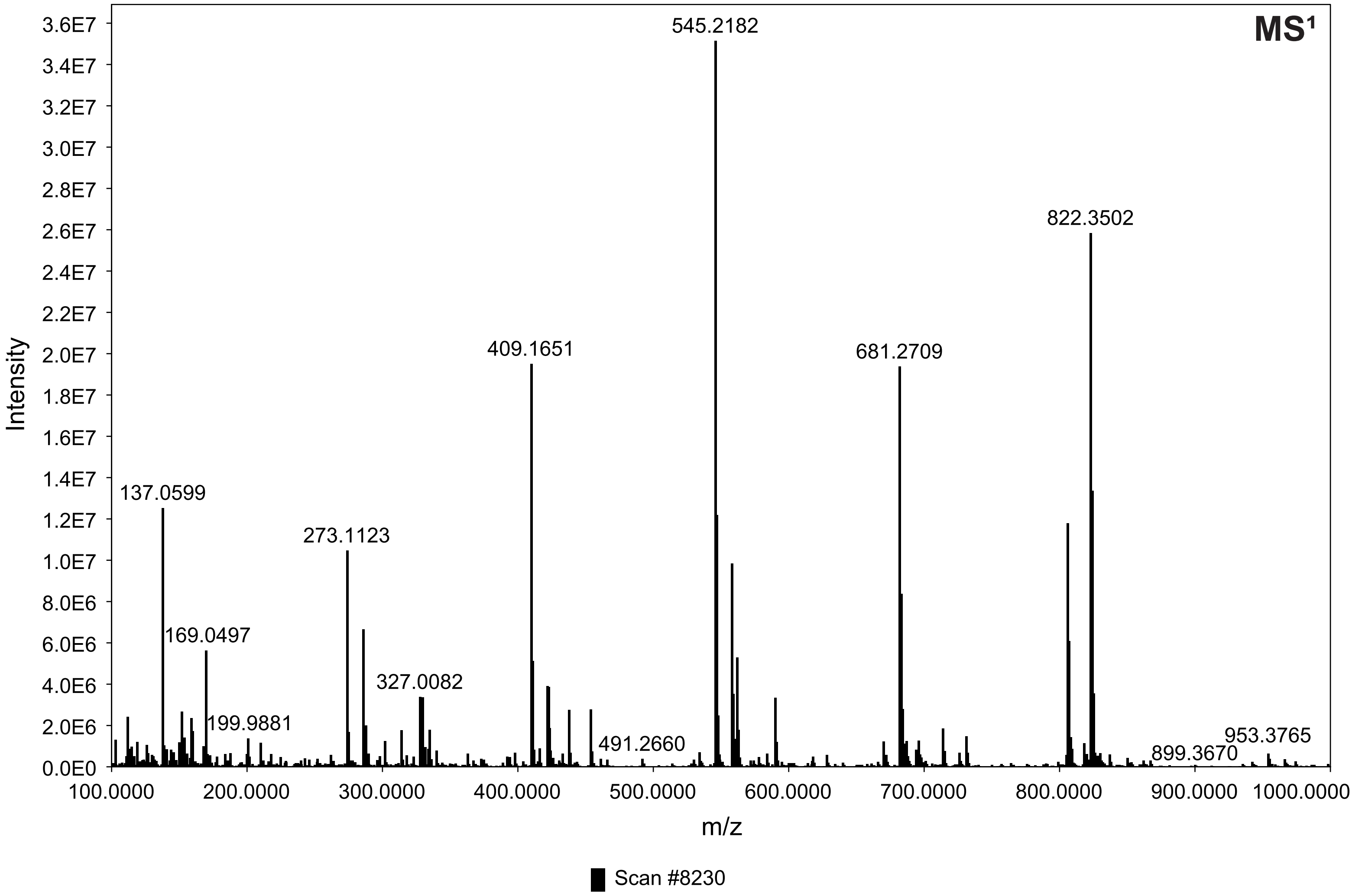

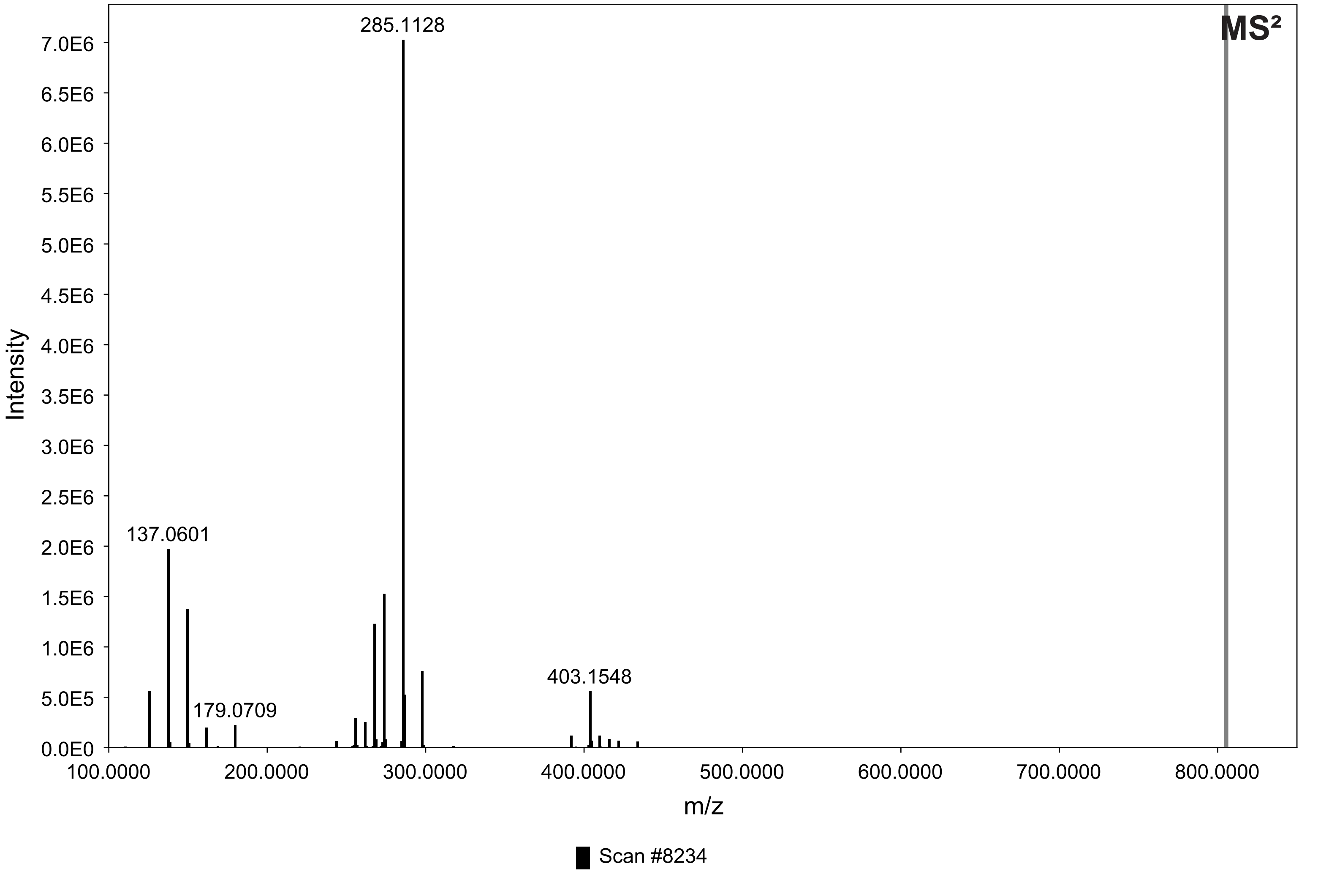

12. LC-MS *m/z* 847 [M-H]⁻; HRMS *m/z* 849.3132 [M+H]⁺

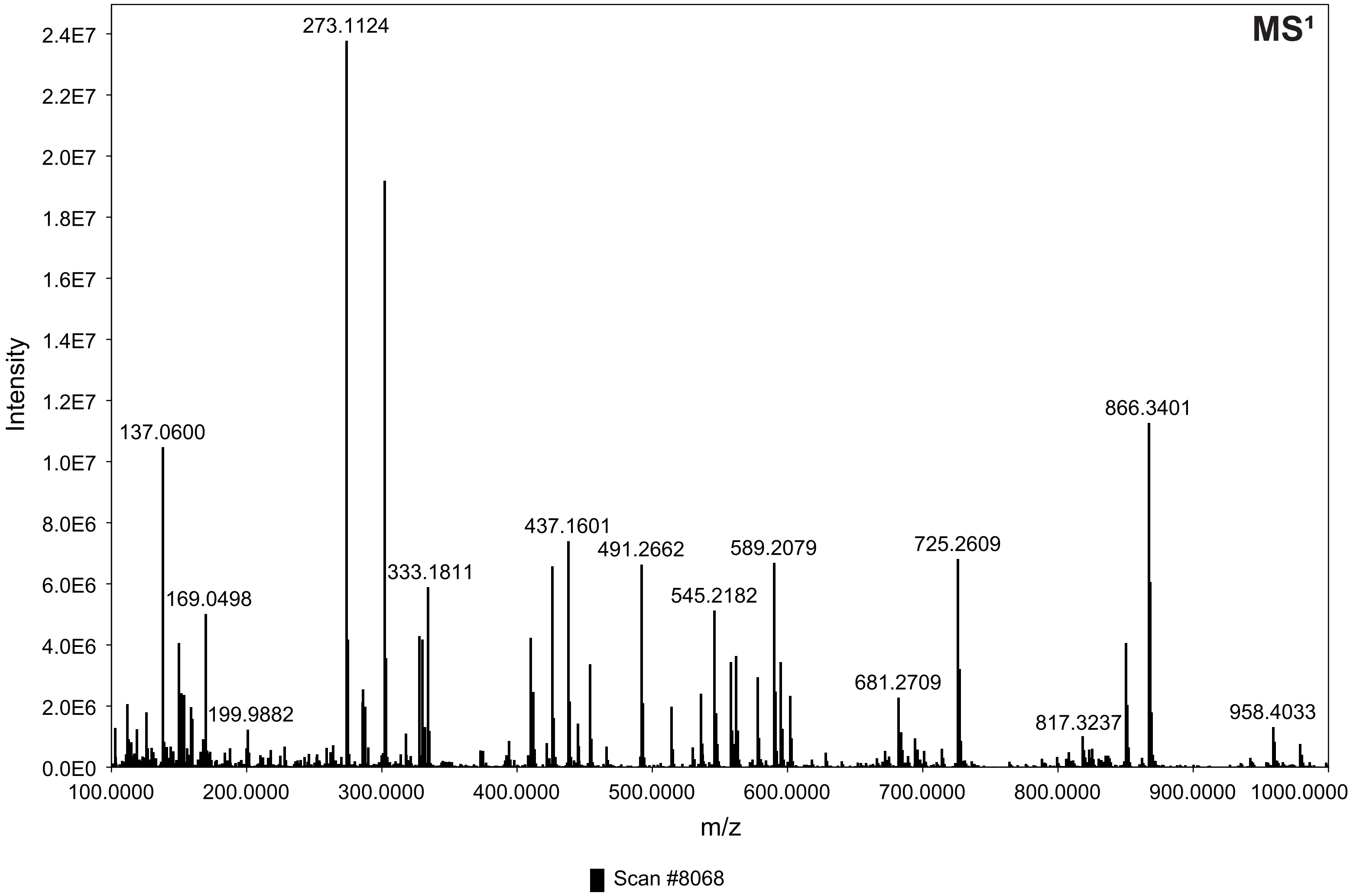

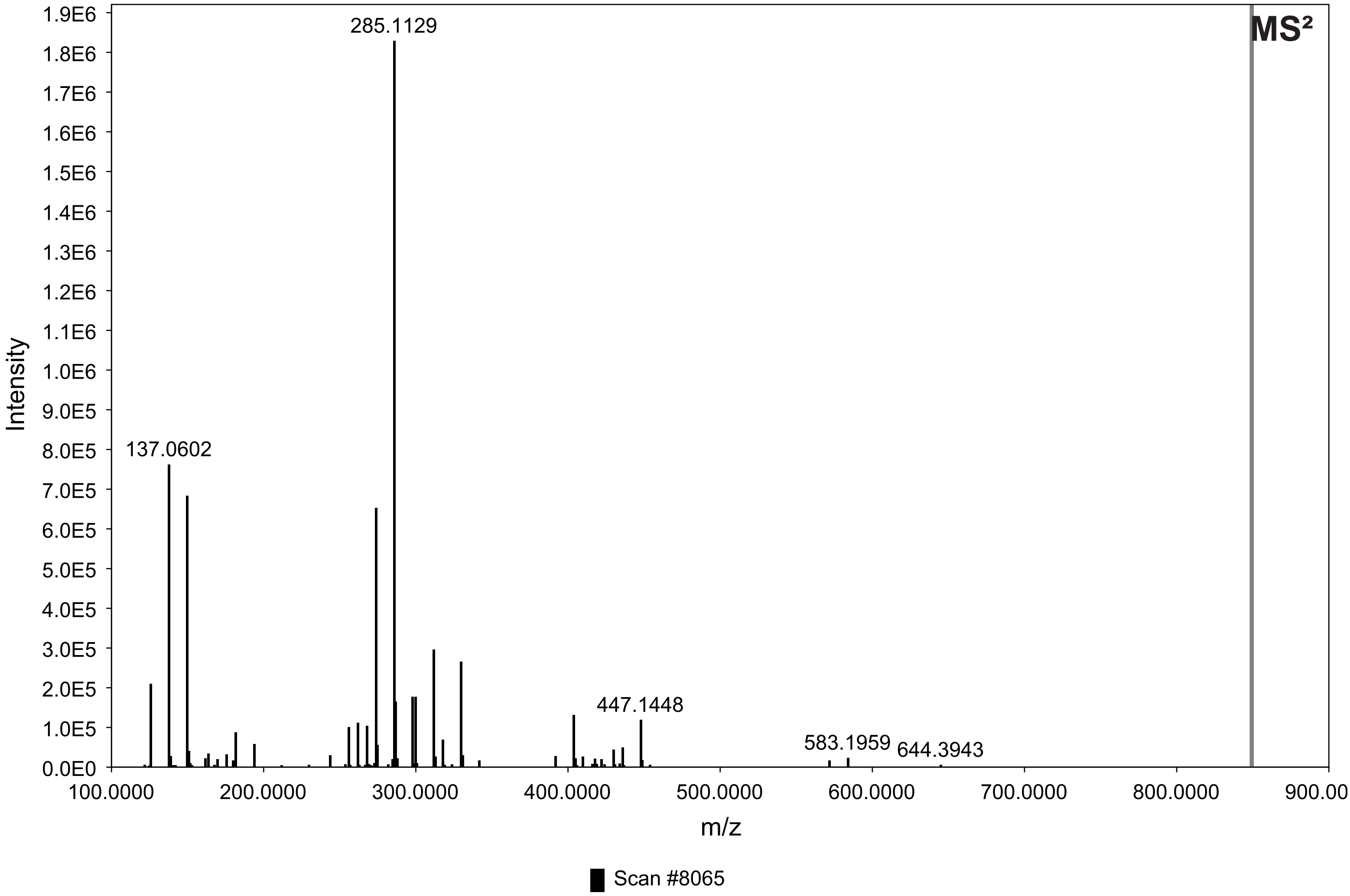

13. LC-MS *m/z* 847 [M-H]⁻; HRMS *m/z* 849.3132 [M+H]⁺

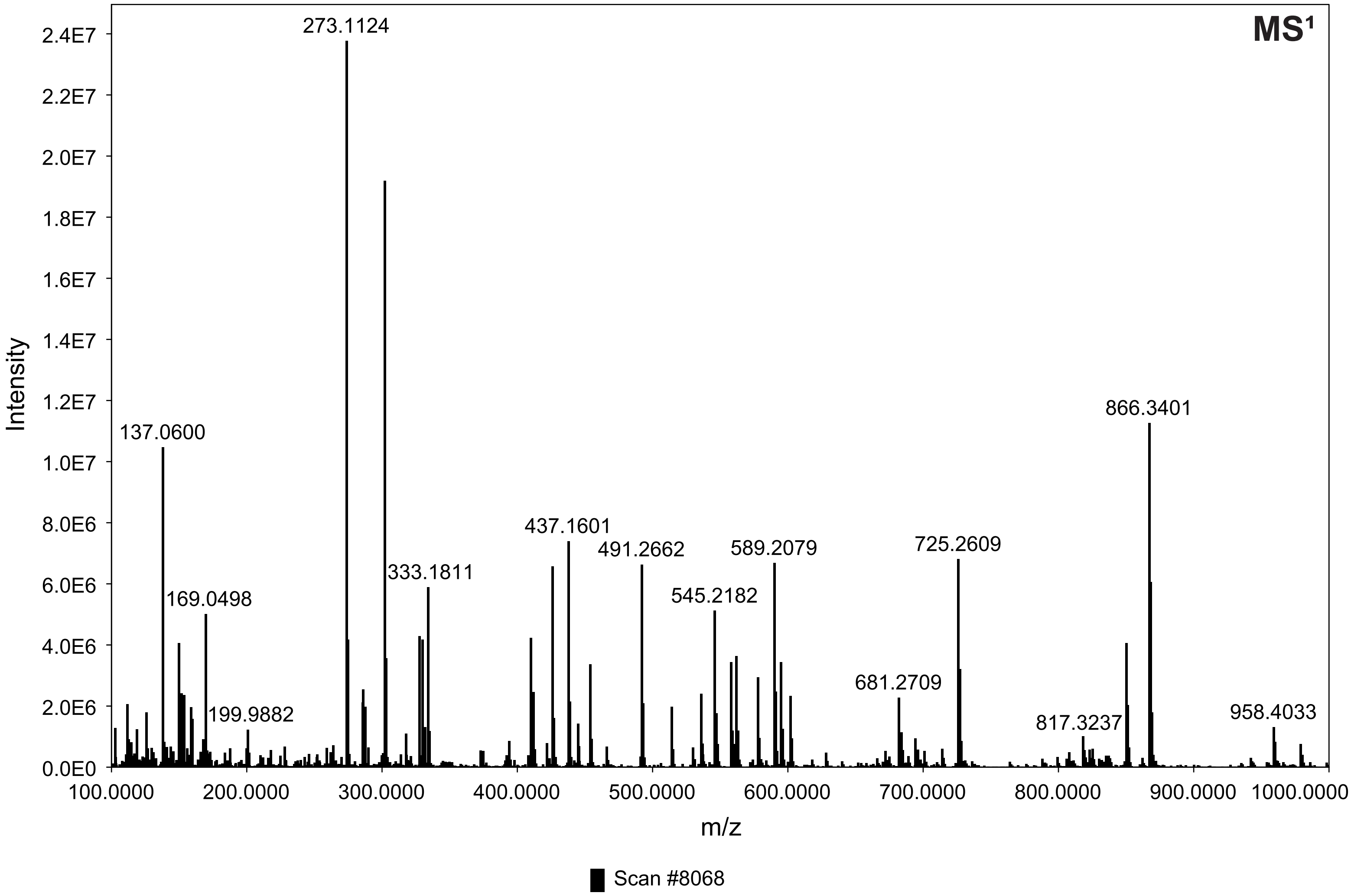

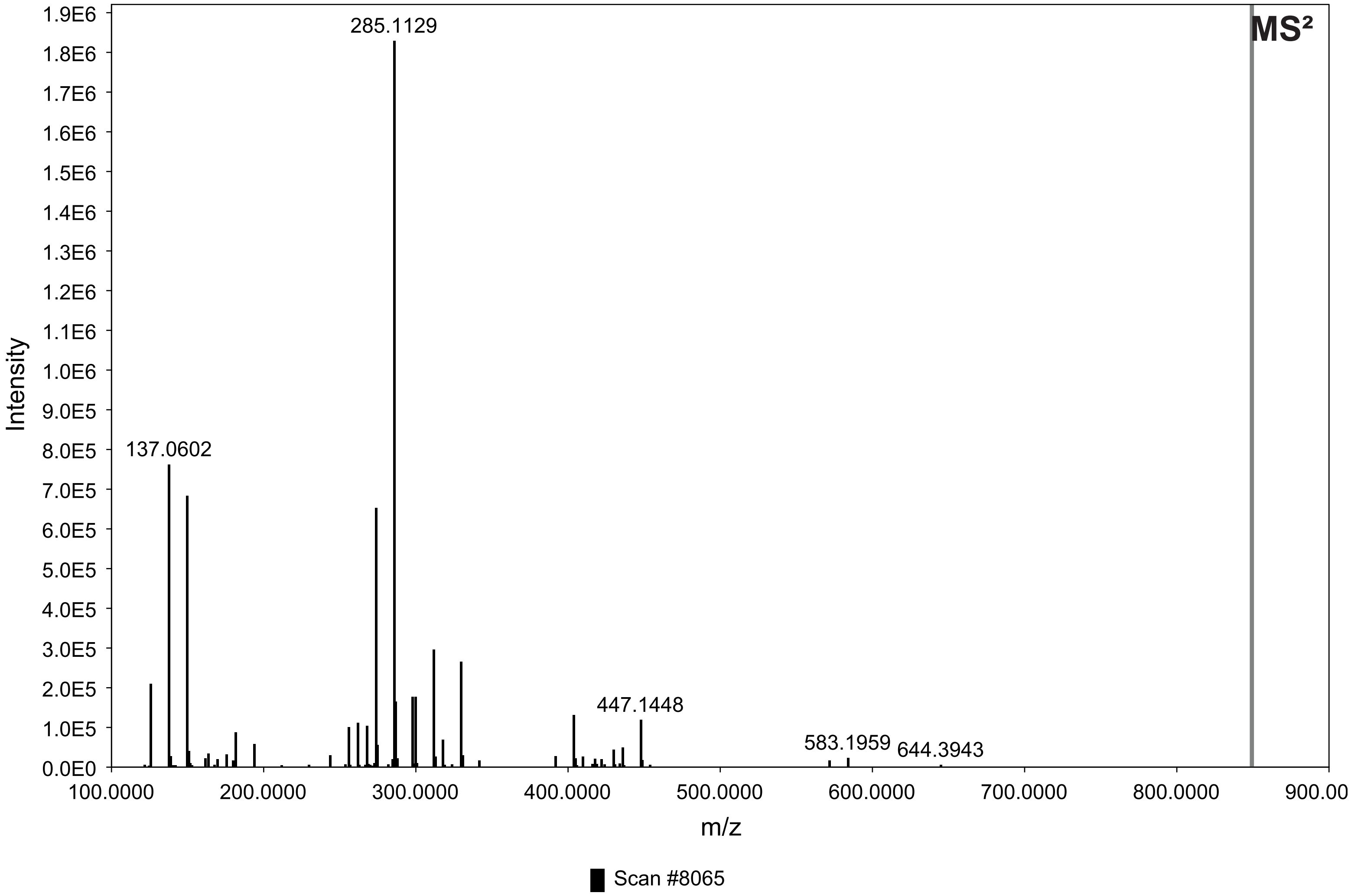

14. LC-MS *m/z* 939 [M-H]$^-$; HRMS *m/z* 941.3759 [M+H]$^+$

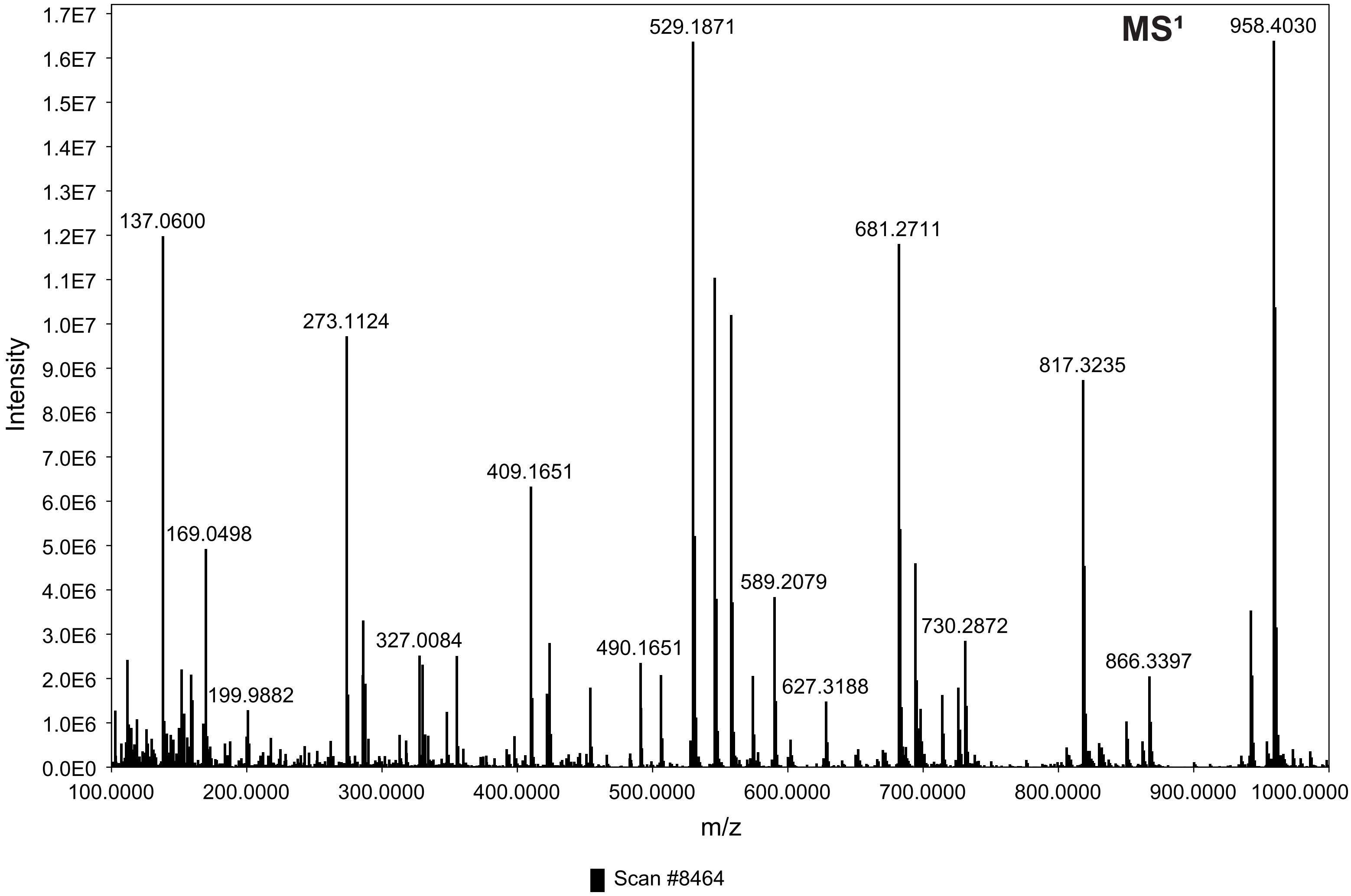

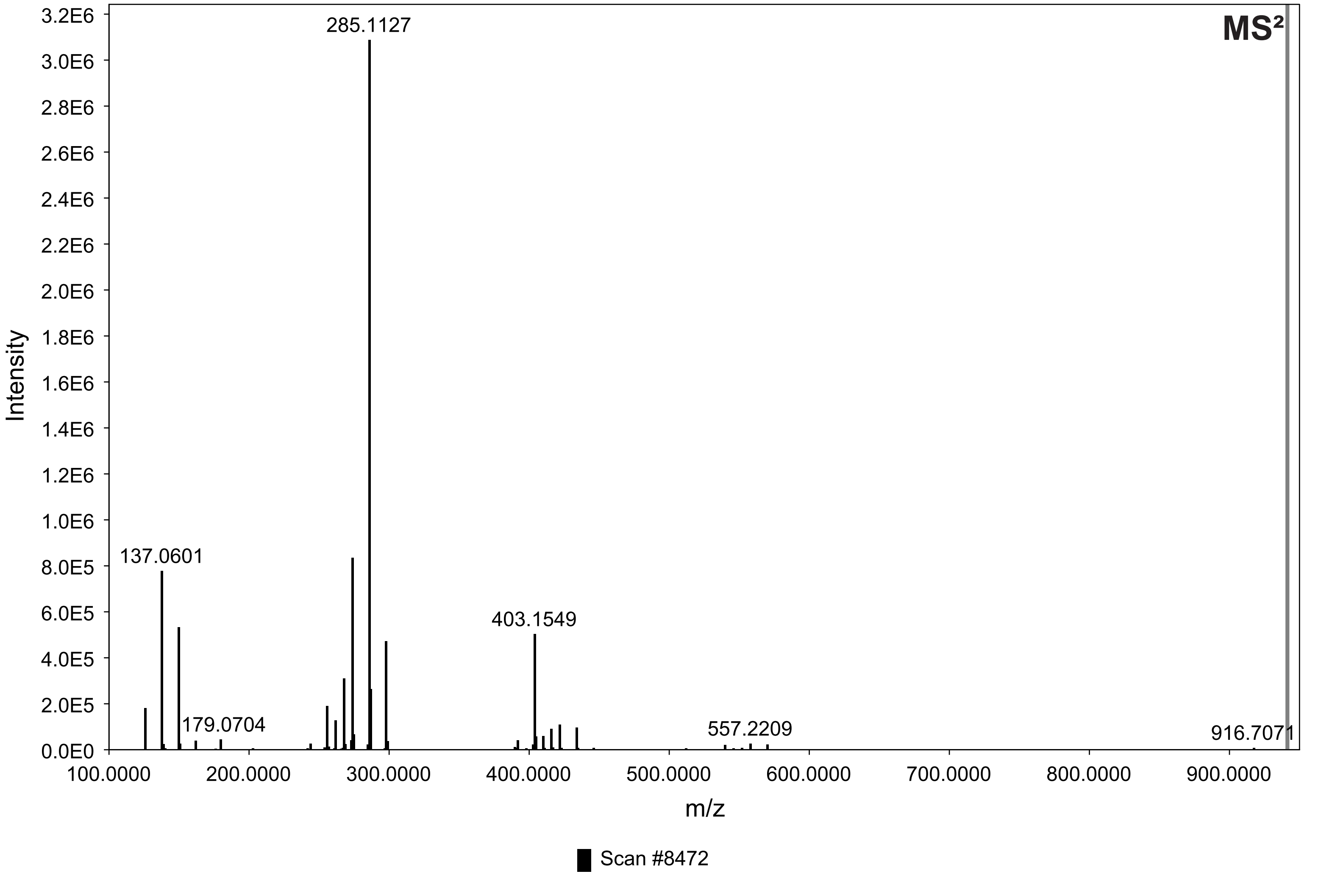

15. LC-MS *m/z* 983 [M-H]⁻; HRMS *m/z* 985.3662 [M+H]⁺

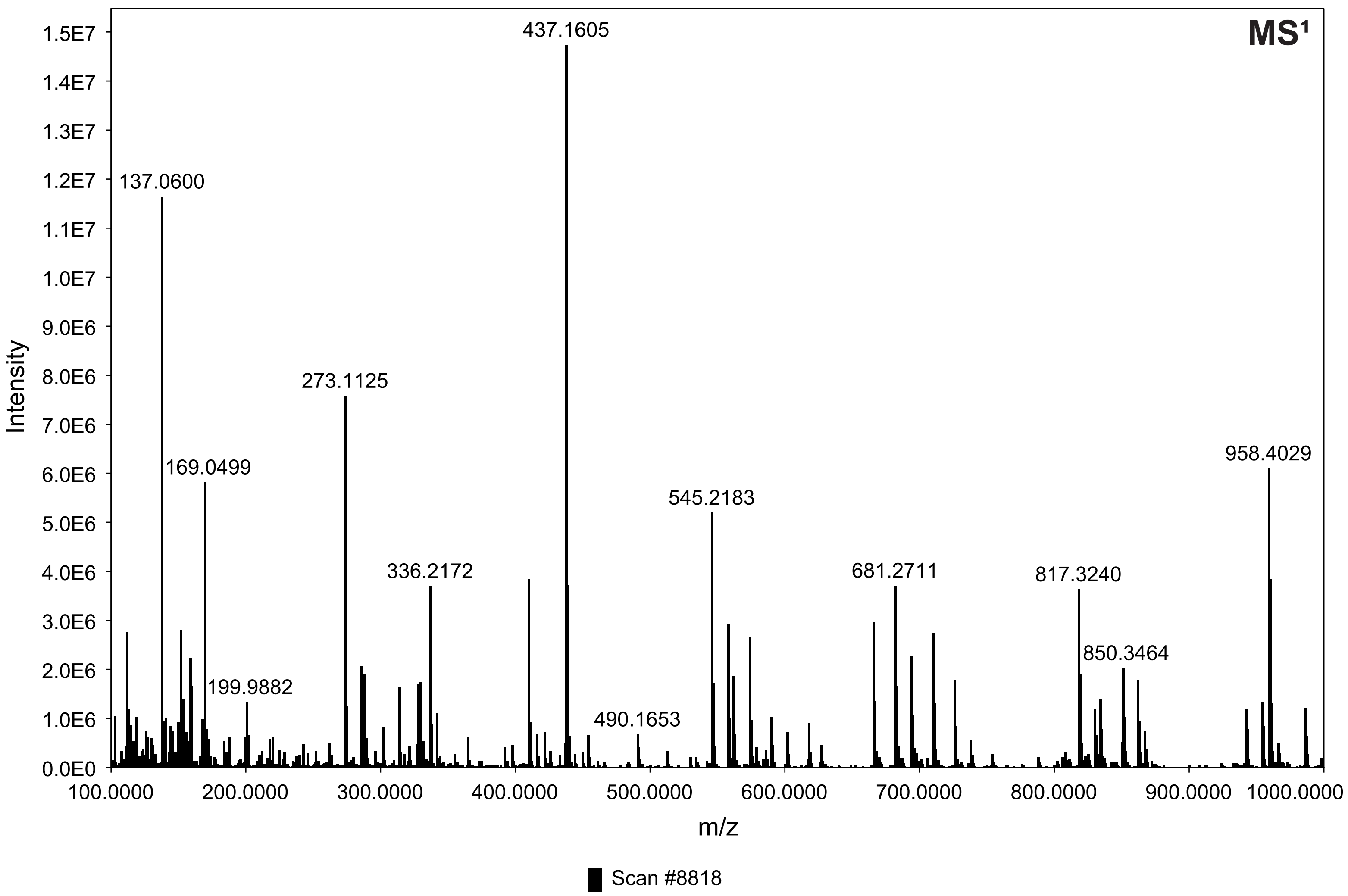

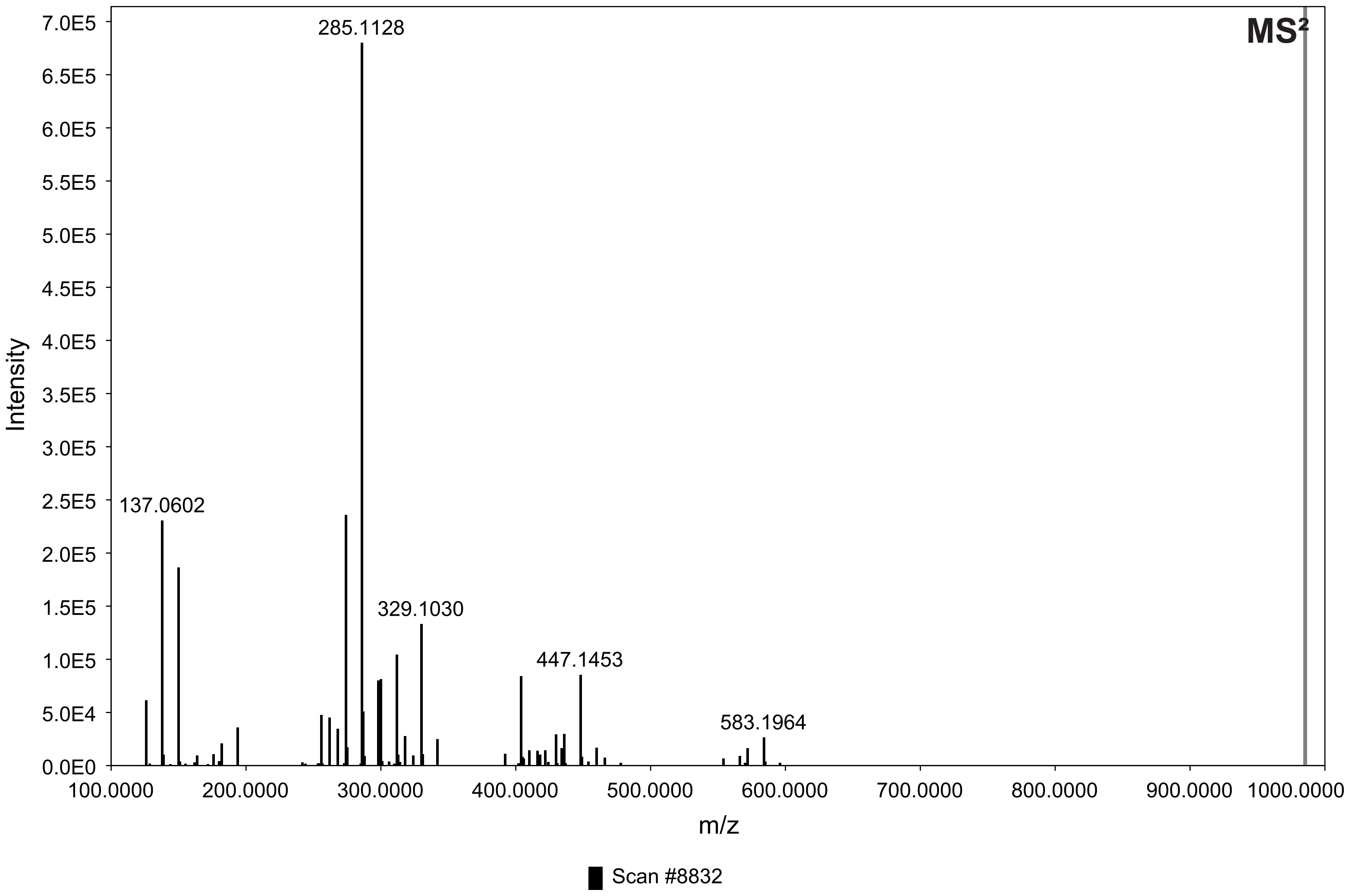

Supplement: Supplementary file 1 [file molecules-29-05576-s001.zip › 20241015_additional-file-S3.pdf]
