# Supplementary material for: Identification and Reconstitution of the First Two Enzymatic Steps for the Biosynthesis of Bioactive Meroterpenoids from Hericium erinaceus (Lion’s Mane Mushroom)
Source: Molecules. 2024 Nov 26;29(23):5576. doi: 10.3390/molecules29235576 (PMC11643632; doi:10.3390/molecules29235576)
Supplement: Supplementary file 1 [file molecules-29-05576-s001.zip › 20241017_additional-file-S1.pdf]

Supplementary Material for

Identification and reconstitution of the first two enzymatic steps for the biosynthesis of bioactive meroterpenoids from *Hericium erinaceus* (Lion's Mane mushroom).

Riccardo Iacovelli[1†*], Fons Poon[1], and Kristina Haslinger[1*]

[1] Department of Chemical and Pharmaceutical Biology, Groningen Research Institute of Pharmacy, University of Groningen, 9713 AV Groningen, The Netherlands

[†] Current address: VTT Technical Research Centre of Finland Ltd, 02150 Espoo, Finland.

*** Correspondence:**

Riccardo Iacovelli

riccardo.iacovelli@vtt.fi

Kristina Haslinger

k.haslinger@rug.nl

Contents

Supplementary tables 2

Supplementary figures 8

Supplementary references 17

Table S1. DNA and protein sequences of the three genes cloned in this work.

Gene name	Nucleotide sequence (5’ → 3’)[a]	Predicted aa sequence[b]
herA<br>(*g019550*)[c]	atgtcctccatcgctgatacgcagcatttcaatgtcccggtcttcgcaggccacggtacaacg<br>gccatcaacacaccccagacgcgtgagcgcgcccttcgcgatgcttcctcgccatcaggct<br>ctattctactctcctcttgctttgatagtttccaagaagagctcgctacattcactgatgaggagc<br>gcaaggctgcaggcgtcgaagctggggacttcgataagcctgagtctctcctctctctatctc<br>aggagcgctacctctccaatccagtcatctctgggatcacgctcttcctcatccaaacgctga<br>ggtacctctccttcgtcgagtcctactcttcgccgtcgtctccgcgcttcgcggccatcctctcc<br>cagaacctcgcacaccagcttggtgttcttggattttcttctggtattctcccagcatcagtcgtc<br>ggcacctcagtttcggcattggagttcatctcaaacgctgtcgaggcattccgcctcgcgttct<br>ggatcggagttcgcgcccaactttatcgcgtcgcagcctttgaatccgcaaatagtctcggag<br>atgatgcggcgctcccatggagccttgtgttcctggggattgggcggcaagaagcagatga<br>ggcagtgcgcaagtaccgtgaatcggtgagtccctcgcgttctagcctcgcgttgtgatgctg<br>acaacccttcgatgcgcgtctgttatgcccctgcttatctcgattcattcctgcatagaatgaaa<br>atgcagagtctctgcacgtgaccgctgtgatggatgagacttgcgtcaccatatctggacgtc<br>ctgacgttctcgctgccttcgcgtcccgacttccgacttctgcgcctccgcacaagacgaccg<br>tagataccctttatcactccccgattcacacaggcacgacacgcgatctggtcctggaagatg<br>tgtcccgccgtgggattcagttcccttcgttctcggacatcaagatccccatccgctccatgca<br>caccggcgagctgctcgattcatctcgcgagggatcgttcgtagaggcggtcgtcgatatgg<br>tcctcacgcagccagtgaactgggatcgcgtcgtcagttcgcttgtgtcagccgcgccagag<br>ggcgaagccgtgcgcttgatcaacgtcgggcctggtgctgggcttactcgcagcatggagc<br>gggcattccccacgcgcaatgcattgtctgtagatctgactgccccggagaagaattccgcc<br>gcgaacaagacgtctcccgttcaggagccgattgctattgttggcatggcggttaacatgccc<br>ggagcaccgagcgtcgagaagctgtgggaggtgctcgagaagggcatcaacactattgcg<br>gaggtaagtgttcttttcctagactccctggacgtacgatctcatagtcctgtcaatcactccag<br>atccctgagcaccggttcaaggtctcagactacaacaaccccgccgatgccaagagtgccc<br>ggtcgatgaaggcacatacgggtaacttcctcgacgatcctgatgcatttgacaacaagttctt<br>caagatctcgccgcgtgaggcacgcagcatggatcctcaaggccgcgtgctgcttcacacc<br>gcgtacgaggcgctggaggattcaggctatgttcccaacgccacgccgacattccagccgg<br>actcgttcgggtgctacatcggttgcgccacgggcgattacgttgagaatctccgaaacgata<br>ttgacgtatattacagcactggtgagagacaaatgaagccaatttcttacctcaactaacgcatt<br>ttctggatagggcactctgcgcgccttcctcagtggtcggatttcatacgctatgaaacttagcg<br>gcccatctgtggtcattgatactgcgtgctcgtcttcgatcatcgccgtgtaccaggcgtgtcgt<br>gcactcatgaacggggactgcactgcagccatggcaggcggtgtcaacgtgatcgctgctc<br>ccgacgtacgtacatttaatcgaaatttctaccggctggactgattgtgcggcgtatcagatgtt<br>catgggtctagatcgtggtcatttcctcagccctacgggccagtgcaaggcgttcgatgactc<br>tgccgatggatactctcgaagcgaaggctgtggaattttcgtcctgaagcgactgtcagatgc<br>catcgctgaggacgacaacatccttggcgtcatccgcagcattgaggttaaccagagcggtc<br>tcgcgagctccatcactcatccgcattcgccgacgcagcagatccttttcaagaaggcgctc<br>gagaagtctggcattgatgcgcgtcgcatcaacgtcgtcgaagctcacggcactggcacgc<br>aggctggcgatcccaacgagctcgatagtattcgcggcgtctttgctgtcggccgtaccccc<br>gcgaacccgcttcacatcacgtccgtgaaggccaacatcgggcatctcgaggctgcgtcgg<br>gctccgcaggcctggcaaagcttctgctcatgcttaagcaccgcacgattcccgcccagatc<br>tcgttgaagaatctgaacccgaagatcgtggctctcgagaaggatcacacggtcatcgacag<br>ggagcacgcgccttggaacccgtcggaagagggactgacgagaattgccatgctcaacaa<br>cttcggcgccgctggttccaatggggctctgttgttggaggaatatgtcccggcgggctcga<br>aggcacctgaggtcgagtctgctgcggccttcatcgttggtctgtcagcgaagacggacga<br>ggcactcaatgccctgcgcatgcgttacatcgagtggctcggcgatgccaagaatgcatcca<br>tctccctcgccgatttcgcatacactgctacagctcgcaggcagttgtacggccagcgtctcg<br>ctgtctctgcaggcaccaaggaggagcttgtcgagaagttgagagccgcgtcgcaggtcgc	MSSIADTQHFNVPVFAGH<br>GTTAINTPQTRERALRDAS<br>SPSGSILLSSCFDSFQEELA<br>TFTDEERKAAGVEAGDFD<br>KPESLLSLSQERYLSNPVIS<br>GITLFLIQTLRYLSFVESYS<br>SPSSPRFAAILSQNLAHQL<br>GVLGFSSGILPASVVGTSV<br>SALEFISNAVEAFRLAFWI<br>GVRAQLYRVAAFESANSL<br>GDDAALPWSLVFLGIGRQ<br>EADEAVRKYRESNENAES<br>LHVTAVMDETCVTISGRP<br>DVLAAFASRLPTSAPPHKT<br>TVDTLYHSPIHTGTTRDLV<br>LEDVSRRGIQFPSFSDIKIPI<br>RSMHTGELLDSSREGSFVE<br>AVVDMVLTQPVNWDRVV<br>SSLVSAAPEGEAVRLINVG<br>PGAGLTRSMERAFPTRNA<br>LSVDLTAPEKNSAANKTSP<br>VQEPIAIVGMAVNMPGAP<br>SVEKLWEVLEKGINTIAEI<br>PEHRFKVSDYNNPADAKS<br>ARSMKAHTGNFLDDPDAF<br>DNKFFKISPREARSMDPQG<br>RVLLHTAYEALEDSGYVP<br>NATPTFQPDSFGCYIGCAT<br>GDYVENLRNDIDVYYSTG<br>TLRAFLSGRISYAMKLSGP<br>SVVIDTACSSSIIAVYQACR<br>ALMNGDCTAAMAGGVNV<br>IAAPDMFMGLDRGHFLSP<br>TGQCKAFDDSADGYSRSE<br>GCGIFVLKRLSDAIAEDDN<br>ILGVIRSIEVNQSGLASSIT<br>HPHSPTQQILFKKALEKSG<br>IDARRINVVEAHGTGTQA<br>GDPNELDSIRGVFAVGRTP<br>ANPLHITSVKANIGHLEAA<br>SGSAGLAKLLLMLKHRTIP<br>AQISLKNLNPKIVALEKDH<br>TVIDREHAPWNPSEEGLTR<br>IAMLNNFGAAGSNGALLL<br>EEYVPAGSKAPEVESAAA<br>FIVGLSAKTDEALNALRM<br>RYIEWLGDAKNASISLADF<br>AYTATARRQLYGQRLAVS

	tgtgactgagcggccagcgaaagttgctttcgtcttctctggccagggcagccagtacctcg	AGTKEELVEKLRAASQVA
	ggatgggatctgccctctacaagaccgtgcctctcttcaagcgtaccgtcgacgagtgccac	VTERPAKVAFVFSGQGSQ
	gctatcttaactgcgtccggcttcccgggcgttctcgccatcatcaatcctgctggtgagacca	YLGMGSALYKTVPLFKRT
	gtggtcttactcagctggaggagttcgaggcatatcaggcggcgatcttctcccttgagtacg	VDECHAILTASGFPGVLAII
	cgctcgccaagttgtggatgtcatggggactggttcccgaggtggtcgtaggccacaggtac	NPAGETSGLTQLEEFEAYQ
	gttcggtttcccgctcgaattgacagtattctgaccgtctatctgtagtttgggcgagtacgcag	AAIFSLEYALAKLWMSWG
	cgcaggtcatcgctggcgtcttgacgctcaagggcgcgctcactctcattgccaaccgcgtc	LVPEVVVGHSLGEYAAQV
	cgcttcatggtcagcaagtgcgccgtggagacgaccggcatgatcgcgataaaccagggct	IAGVLTLKGALTLIANRVR
	ccgaggcggttgcgaagctgctggcagcttcgatggacttccccgacacttctgttgcatgct	FMVSKCAVETTGMIAINQ
	ttaacagcaacactgactgcgtcgtttcgggccctattcctcagctcaaggcactgaaggcac	GSEAVAKLLAASMDFPDT
	acctcgacagtgaagtacgctgcaagaacgtcctcttgacggtgccgttcgggtaccatagc	SVACFNSNTDCVVSGPIPQ
	tccgccatgcacccccttctcgacgacctttccactattgctaagcatgttaccatccgcgcgc	LKALKAHLDSEVRCKNVL
	ccactattccgattatctccaatgtcaccggtgaggtcgttatgcccggcgatgagggcgtatt	LTVPFGYHSSAMHPLLDD
	cgactcggagtactactctcgccactgcgcgcagccagtactcttcgagaagggcctcactt	LSTIAKHVTIRAPTIPIISNV
	cactcgcggctattccggagcttgcgaacatcgacgcatggatcgagattggtccccacagc	TGEVVMPGDEGVFDSEYY
	atcacgcttcccatgttcaaggttcacccgtcgatctcgaagagcaccatgctcttgggctccc	SRHCAQPVLFEKGLTSLA
	tgaagaagaaccaggacccgtgggcaatcgtctcttcgactttggctcagctgtacacgtccc	AIPELANIDAWIEIGPHSIT
	cgattcaactcaggtggcgtgaggtctttgcccatgtctcttctccttcgacgctttccctcccgt	LPMFKVHPSISKSTMLLGS
	cgtacccccttcacgaagtccaagttctgggtctcgttcaaggaggaggcacctggcgcggtt	LKKNQDPWAIVSSTLAQL
	gtatcggcgtccacctccgtcccgctcgcgaagcacgtcgatttagtcaataacttctcgatgc	YTSPIQLRWREVFAHVSSP
	tatactcctgggcccagttcccgtcggcggcgaaccagcgcgtcgcaattttcgagacgccc	STLSLPSYPFTKSKFWVSF
	atctcgcagctcgggaagtccatcaagggccacagcgtcggagaccacccgctctgcccc	KEEAPGAVVSASTSVPLA
	gcgtccgtgtaccacgagctcgcacttgcaggtatcgagatggctaggtcccatctgcacctt	KHVDLVNNFSMLYSWAQ
	aagattgacgactgcttcgtcatgcttcgcgacatcgactacgcgaagccgctggtctacaac	FPSAANQRVAIFETPISQLG
	gagcacgttgcccggatggtcaggacatcgatcacacttgatgttgatggctcgggctctttc	KSIKGHSVGDHPLCPASVY
	agcgttggctcgcaggtagatggctcgcccgaggaggtccattgcttcgggaagttcaagc	HELALAGIEMARSHLHLKI
	accagtcgacatcgaaggcctcaaccaagttcgctcgcgttcttcccatcgtaactcgtcaaat	DDCFVMLRDIDYAKPLVY
	cagctccgtctcgtcgcctaacgatggtttcgcggagacgttctctactcgcacggcatatga	NEHVARMVRTSITLDVDG
	ggtcatcttcccgcgcgtggtcgactacgcaaaggagtaccacacgatgaagacgctcacc	SGSFSVGSQVDGSPEEVHC
	gtcgcctcgaacggtatggaagcatacgcgatcgtccagctgcctcgcgatcacgaccgca	FGKFKHQSTSKASTKFAR
	gcaagtttgtcgtccaccctgtcttcatggacaccatgcttcacgttgccggcttcgtcgcgaa	VLPIVTRQISSVSSPNDGFA
	catgcagggcggagtcaacgatgcctacatctgcagcaaggtcgacacagtgaaggcgat	ETFSTRTAYEVIFPRVVDY
	cccggatttgatcgacaacgacgcggagtacggcgtgctgatcagcaacgcctgggtcgc	AKEYHTMKTLTVASNGM
	ggatgagggcgtcatgcttgcggaggcctacgccgtccagttgaagtctccaggtaagatc	EAYAIVQLPRDHDRSKFV
	gtcgcgcacctcaagggcatgcacttccgcaaggtgcgcctcaatagcctgaagcgcggtc	VHPVFMDTMLHVAGFVA
	tcgcgatggccgcgggcactagctcggcgcatgctgcgccgaagcgggcggaggcgcct	NMQGGVNDAYICSKVDT
	gtgaagaaggctgcgcctgcttccccgatgtcctccaaggtcgtttcctcaatcaccttcgtcg	VKAIPDLIDNDAEYGVLIS
	agccgcctcgagatgcagcacctactatcgacgtcctcgcggaggtcacgcgcatcgtggc	NAWVADEGVMLAEAYAV
	cgagacttgcgacatcactgcatccacgatccagcccgatggggacctcgaggcgtacgg	QLKSPGKIVAHLKGMHFR
	cgtggattcgctgatgtccattgagatcttcaccaagatgcagagcgccttcgctagtgctgac	KVRLNSLKRGLAMAAGTS
	cttgacgcgaacgtcctttcctcgtgccgcaatgttgcccagatcgtcgctgaggtgtcgtcca	SAHAAPKRAEAPVKKAAP
	agttctcccaggaggactctggcccatccacgccgcgcacactcgtcactgacgagaagctt	ASPMSSKVVSSITFVEPPR
	ggcgagcccagcgtcatcgcgaacttcgacgaggtcgacgtcaagccgctgctggcgtcc	DAAPTIDVLAEVTRIVAET
	gtcctcggcatcggcctccaggagatcaccgacgacgcggacttcgaatcgctcggcttag	CDITASTIQPDGDLEAYGV
	actcgctgacatccatcgaggcgcattcggccctgcagagcgagtactcgctgacgctgcc	DSLMSIEIFTKMQSAFASA
	gaccaccctgttcgagacatacacaacggcgaaggccgtcaacgccttcctcacatcgcag	DLDANVLSSCRNVAQIVA
	ctgcgtcctcggggcaaggccgtcgaggtcgttaaggaggccgaggtgcacccttcggact	EVSSKFSQEDSGPSTPRTL
	acaaggactcggacatcgcgaccgccgcgaaggtcgcgcagatcgtcgacggcaatctca	VTDEKLGEPSVIANFDEVD
	acccgctcgtcaccgcgctgcgcctcgactcggttcccgtcggtgcgcagaaggcgaaga	VKPLLASVLGIGLQEITDD
	cacccggccgcgcgcccttattcctgattcacgatggtagcggccttgtgaactacatccagc	ADFESLGLDSLTSIEAHSA
	gcctctccccgcttgaccgcgacatttggggcattcataatccccacttcatcaccagccagc	LQSEYSLTLPTTLFETYTT
	cgtgggagagtgtcgtgtcgatggctgcggagtactctgagttcgcgacgaagacgacctct	AKAVNAFLTSQLRPRGKA
	gagcctcttatccttggtggtgagtacccctctatcgctaccttattcttgctttgttgataattgttt	VEVVKEAEVHPSDYKDSD
	atctcaggctggtcgttcggtggtgtcgttgccttcgaagctgcgcgtcagctcatgaagaag	IATAAKVAQIVDGNLNPL
	ggcgttgccgtgaagggcgtcctcctcatcgactcccctactcctctcgcccacgttccgctct	VTALRLDSVPVGAQKAKT
	ccgacgccctcctcgagtccgtcgcgaagctggatggccgcgtcgacacgcaggttggca	PGRAPLFLIHDGSGLVNYI
	agctcgtgaagacgcagttccagatgaactcgcgtatgctcggccgctatgacccgcttgcc	QRLSPLDRDIWGIHNPHFI
	gcgggcgggccgtttccctcgatcgtcctcctccgctcgagagagggcttcaaacccgccg	TSQPWESVVSMAAEYSEF

	gcgtcgccgacgtgccgaagtggcttgcggacaggagcgatgcgcagctggccatatccg<br>ggtgggagcgcgtcgtgggcacgccgatcaaggccatcgacatccccggcaaccatttcc<br>agcctttccacacctctaatgtaagtacttgctgctgtctcttgagacgtcttgtcgctgaccata<br>gatttttagatcgaagaagtttctcgtcgcattgctgaggggtgcgcacacctcgagagccttg<br>ctgcttgaaggccgtactgtacactattcccttcttttgtatcatgcgtcatgacatccgacatgtt<br>tctcctgcatcagtagaggttcacgattcccattgtattttactgcgctgctgctttacatgctgct<br>ccttgttacgattttcacgtagtctctag	ATKTTSEPLILGGWSFGGV<br>VAFEAARQLMKKGVAVK<br>GVLLIDSPTPLAHVPLSDA<br>LLESVAKLDGRVDTQVGK<br>LVKTQFQMNSRMLGRYD<br>PLAAGGPFPSIVLLRSREGF<br>KPAGVADVPKWLADRSD<br>AQLAISGWERVVGTPIKAI<br>DIPGNHFQPFHTSNIEEVSR<br>RIAEGCAHLESLAA*
herB<br>(*g019600*)	atgccgattcccgtgaatactttcgttcgcccacggctcaatctgggatacggccaagacagc<br>agcgaaggcgtcaactcactcccagagttgatcgccttcaatgccgagcataatcccggcca<br>tgtcttcggcttgcagattcgcgctggcgagaacatttcgccttgcaaaatgacattcgcagaa<br>cttcatgccgccgttgagcgtgcgtcggcatggctcatggcatctggtgccaccgcggggc<br>ggacgtcgcgggacaccaaggtcgctcccgtcgctattttactggggagcgatattggaatc<br>ttcatctatatggctgcattattgcggattggcacgccggtaattattctgtacctttcggactatc<br>tttaaaggtgagctaacgacgaactttttcaggtccttctgctttccgcccgccttaccccaatc<br>gccatcgcacatctcatcaaggcgacatctccttcgacgatcctcataaacgctcaagtctctc<br>gatccgccaatgagaccgttgatcttttggcgactgatgacagcgccttcaccagaccgcaat<br>tcctccatgctttgggttacgaggatttcatcagcggcgaacatcccgacctgcaaaatctttc<br>catccctcccgtctacgacgctttcaagtatgaggatctcgacgcgatcatcatgcactcatcc<br>ggcaccaccggtcttcccaagcccatctaccacgcgcaggcgtaccttctcatctacgctgg<br>ctgccactgcatccccgagtcaagagagccgtcgcatttcaacgtttctagcttgcctctctatc<br>atgtgcgtccgcagttttgcgcttttccacccgattctgacgttcaatattccacagggcttcgg<br>attattagcaccgtcgctttccctctctatcggcctcccattcgtcttacccccctgcctctatcatt<br>ccgacggcacggacggtattgaacagtctagagttaacgcgcgcacggtctatgctgtccgt<br>cccttccatattggaggatattgtcagactgcctggtgccgctggcttggaagcgctcaagaa<br>actggacttcattgccatcggaggcgctcccatgaaagaagccgtcgcacaagaacttgtttc<br>gaacggcgtcaatctgctcaatcactggggtgcgtctttctgatgttcttaaaatatgtcgcagc<br>atgttgactggcttgttcaatacaggagccacggagattggtgccatcgcacctgttcagcgc<br>ccgcctctgggatacgattggcattacctcatccctcgcactgacattggcctcgaggtgatc<br>cagctcgatgacgctggccgcacttaccgccttatcgggcgggctcccggctggccggag<br>ccattcgtcgtccaagatcttctcgaagtacacccatctgatccgacgcagttcaagatccttg<br>gacgcgccgacgatctgatcgtactcgccacgggcgaaaaagtccgccccacgagcatgg<br>aagctgccattgcagagcaccccgacgtaaaggccgtcctggcgttcggagatggccagtt<br>ctcgctcggacttttggtcgagctggcgtcttcaaagtccgggctggatctttctctgcctgcca<br>atgtggatgccgtgctggagacgatggagccccacctggagcgcggcaactcgttcatgga<br>caagcatgcaaaaatcacgaaggatatgattgtactcactcagcccgagattaagccgctcgt<br>gcgcaccgacaagggcagcctggcgcgaaaggcgacgttctttgcgttcgagaaggagat<br>caaagagtgctacgaacgtgcggatgtggcgcgcgctgtacccttcccactctacagcgtcg<br>acgagggtgcctctctcctgtcctcgttgcgggctcttgttgggtccaccctcggaattgatga<br>catcggcaccttcagcgacgagtccgatttcttcgaggctggcatggacagtctgcaggcca<br>gccgtctacgtcgtgccattcagaacggcctccgcattaccaaagacatttcaggtcccgtcc<br>cggaactcgctcctgacttcgtcttcgagcactcctccgtgaaaaggatatgcaacgtcatgg<br>ctcacatcatgctcgggacttatgaagccgacgccggcatggacaaggaggagcgaagga<br>tagctgctatgggcgacatggtccaacgctacgcgaaggagcttacctggtacgccgaaga<br>cgcgcttctggctcgcgaagcaagggccgcatcttctcacaagcgtgcctcgacgtcgaagt<br>ccactgtgctgttgacaggctctacgggcagtctgggttgcatgctcttggcacagctcgccg<br>gcgacccggcgtcgccaagatcatctgtttgaatcggccacagcagggcggtatcgacatt<br>cggaagcgacaggcagatgcgatgaagaagcgcggcgcccatatcgacgctgaggggtg<br>ggataaggtcgtcatctacgaggcggacataagccgcgcagacttcggtctcagtgacaac<br>gaatttgaagaggtaagggaccatctctgtctctctgtcttctctgttcatttatgattctccccag<br>ctgttagaggtgacgcacatcattcacaacgcgtggcctgtcaacttcaatcgcacgctggct<br>tcgtttgattcccatgtcagagccctttgcaaccttgcgcggctatcccttctcagtgcagcgaa<br>gttccctacggatcgtcgaccgcggcgcatcttgttcgcatcgtcgatcgctgtggtcggccg<br>gttcccgctccttcatccggaggggcccttcgacgtgccagagactccccttgatgctgcga	MPIPVNTFVRPRLNLGYG<br>QDSSEGVNSLPELIAFNAE<br>HNPGHVFGLQIRAGENISP<br>CKMTFAELHAAVERASA<br>WLMASGATAGRTSRDTK<br>VAPVAILLGSDIGIFIYMAA<br>LLRIGTPVLLLSARLTPIAI<br>AHLIKATSPSTILINAQVSR<br>SANETVDLLATDDSAFTRP<br>QFLHALGYEDFISGEHPDL<br>QNLSIPPVYDAFKYEDLDA<br>IIMHSSGTTGLPKPIYHAQ<br>AYLLIYAGCHCIPESREPS<br>HFNVSSLPLYHGFGLLAPS<br>LSLSIGLPFVLPPASIIPTAR<br>TVLNSLELTRARSMLSVPS<br>ILEDIVRLPGAAGLEALKK<br>LDFIAIGGAPMKEAVAQEL<br>VSNGVNLLNHWGATEIGA<br>IAPVQRPPLGYDWHYLIPR<br>TDIGLEVIQLDDAGRTYRL<br>IGRAPGWPEPFVVQDLLE<br>VHPSDPTQFKILGRADDLI<br>VLATGEKVRPTSMEAAIA<br>EHPDVKAVLAFGDGQFSL<br>GLLVELASSKSGLDLSLPA<br>NVDAVLETMEPHLERGNS<br>FMDKHAKITKDMIVLTQP<br>EIKPLVRTDKGSLARKATF<br>FAFEKEIKECYERADVAR<br>AVPFPLYSVDEGASLLSSL<br>RALVGSTLGIDDIGTFSDE<br>SDFFEAGMDSLQASRLRR<br>AIQNGLRITKDISGPVPELA<br>PDFVFEHSSVKRICNVMA<br>HIMLGTYEADAGMDKEER<br>RIAAMGDMVQRYAKELT<br>WYAEDALLAREARAASSH<br>KRASTSKSTVLLTGSTGSL<br>GCMLLAQLAGDPGVAKII<br>CLNRPQQGGIDIRKRQAD<br>AMKKRGAHIDAEGWDKV<br>VIYEADISRADFGLSDNEF<br>EELLEVTHIIHNAWPVNFN<br>RTLASFDSHVRALCNLAR<br>LSLLSAAKFPTDRRPRRILF

	acaccgctgagttcggataccctgaggcaaaatgggtgtgcgagcgggtattgcaaacattc<br>tcagacctgtacggcaagcctggccatcggcaggaggaggcccttgtccagacgtcaagc<br>gtccgcatcggacaaatgacgggtcctgaaggctctggcgcgtggaacgagaacgagcac<br>ttccccatcattgtgcgcacctcgcagaagctcagggcattgcctgacattgacggcgtgagt<br>atactctccctgcgttgctttcaagtgactcataattgctccagtcattgtcatggatgcctgtca<br>accgtgcggggtcggccatcgtggatttcctcttctcgaagaacttccgtccaatctaccacat<br>ggagaatccgtcgcgccagtcgtggtcaggtcttctagagaaccttgcatcagttcttggcga<br>tagagacgggcctctgcctgtgatcccgtacgacaagtggctgcagcgcgtccgcgacctc<br>ggctctgacccggagaagaacgtcgcgtacaaggtcatggacttcttggagaatgacttcgt<br>gcggatggcgtccgggagcgtaatcttgggcaccgacgtcgcgaaacgcgattcactgac<br>gatggtgaagagcgtcgcactggacaaaaagcacctggctgagtatgttgcttattggagga<br>gtgttggtgcactgcagtag	ASSIAVVGRFPLLHPEGPF<br>DVPETPLDAANTAEFGYP<br>EAKWVCERVLQTFSDLYG<br>KPGHRQEEALVQTSSVRIG<br>QMTGPEGSGAWNENEHFP<br>IIVRTSQKLRALPDIDGSLS<br>WMPVNRAGSAIVDFLFSK<br>NFRPIYHMENPSRQSWSG<br>LLENLASVLGDRDGPLPVI<br>PYDKWLQRVRDLGSDPEK<br>NVAYKVMDFLENDFVRM<br>ASGSVILGTDVAKRDSLT<br>MVKSVALDKKHLAEYVA<br>YWRSVGALQ*
g074890	atggccaacgagaagacgcccctcagagagcccatccagctcaagaaaaagacgctgcct<br>ttcccttatggcctcttccccgcatcgctgcgtccatatgctgagctcatgcggttgcacagagt<br>acgcaccacccttctctctcctcacaggcgcgtctgacatttattcatcagccttccgggacca<br>tcatgattttctggccgtatggtatggcgtctgttcgccatcatgcatcggaagctcaatggcat<br>ttttcattttagcgtttggcgcgacgatggccgcttaccagacgggcttccctgtcaagcagta<br>ctgggcagagcttggcaagttcctcatcgctgcgttcttcgtgcgtagcgcgggctgcaccat<br>caacgacattgccgaccgggagtttgacgctggcgtcgggtgggtccttttaaatattcttttgtc<br>cagaataatattgacgccctcttggtagagaggaccaagagtcggccattggctagcggac<br>gcgtcaccgtgtttgcagcatatgtgttctgcattctccaatggatctgcgcgatcttcgttttctt<br>gccctacaactcgacaacgtacgtacaaccgtccgtgcttcacttggcctgctttggctgacg<br>gcgtcatgcacaccacccagaatgatcgcagcaattctgcaatgcatcccgatgtcgacggc<br>ttatccgtatatgaagcgcatcacatactggccccaggcgtggctcggtctgacgatgtcgat<br>gggcatctttatcggctgggccgcgatcgcagagacgccgaattggctcctgctcggttcgtt<br>catgctcggcttcgtcgcttggacactgcacttcggtacgcactttctctagcctccgtagccg<br>cgcgcttactgacgcgtccattccatgcgcgcctagacaccatatacgcctgccaagaccgc<br>aaagacgacatcaaggtcggcgtcaagtcgaccgcagtgatcctgggtgacctcgtcatcc<br>cgttcgcgatggtgtgctcgacgacgttcgtcggtgcgctcgcgttcgcggggtacctcaac<br>ggcgagacgaaggcctactacttcgtcaccgtcgcgggcaccgcggcgcacttcgtgtgg<br>cagcttgcgaccgtcgacctcgaggacggggatagctgcagccgtatgtatttcctcatatgt<br>aatgacatagcgcttatctgtcaaacagtcaactttacacgcaatggacaacttggatgggtcc<br>tctggggcggaatggcaatcgactatctgctcaagatgggcgtcattcagctcggcgaggg<br>ccggcttagcattgttgtatga	MANEKTPLREPIQLKKKTL<br>PFPYGLFPASLRPYAELMR<br>LHRPSGTIMIFWPYAFGAT<br>MAAYQTGFPVKQYWAEL<br>GKFLIAAFFVRSAGCTINDI<br>ADREFDAGVERTKSRPLA<br>SGRVTVFAAYVFCILQWIC<br>AIFVFLPYNSTTMIAAILQC<br>IPMSTAYPYMKRITYWPQ<br>AWLGLTMSMGIFIGWAAI<br>AETPNWLLLGSFMLGFVA<br>WTLHFDTIYACQDRKDDI<br>KVGVKSTAVILGDLVIPFA<br>MVCSTTFVGALAFAGYLN<br>GETKAYYFVTVAGTAAHF<br>VWQLATVDLEDGDSCSLN<br>FTRNGQLGWVLWGGMAI<br>DYLLKMGVIQLGEGRLSIV<br>V*

a. Predicted introns are highlighted in grey.

b. Predicted aa sequences are based on structural and functional annotation performed with the GenSAS v6.0 webtools (additional file 4).

c. *herA* was cloned prior to re-annotation of the genome, as predicted by fungiSMASH (gene 34, Figure S3), and therefore contains an extra stretch of 155 bp at its 3' end, highlighted in yellow in the nt sequence. The stop codon of the correctly annotated *g019550* is underlined and bold.

Table S2. Genomic location of the hericenone BGC and the non-clustered *g074890* gene encoding a putative prenyltransferase.

Organism & genome assembly (GenBank No.)	Scaffold/contig (GenBank No.)	BGC genes	Location of BGC genes (nt)
Hericium erinaceus strain 0605 (GCA_016906435.1)	contig 12 (JABWEG010000012.1)	HE-BGC4.1 (fungiSMASH)	100,553 – 171,961 (total: 71,409 nt)
	contig 5 (JABWEG010000005.1)	*g074890*	960,050 – 961,393 (total: 1,344 nt)

Table S3. Comparison between the NRPS-like CAR gene preliminary annotated with Prodigal integrated into fungiSMASH or annotated with GenSAS v6.0 pipeline prior to fungiSMASH analysis.

Gene ID (annotation tool)	Location on contig (nt)	Length (nt)	Predicted protein length (aa)
Gene 39 (fungiSMASH)	147,813 – 152,276	4,463 (2,889 w/o introns)	962
Gene *g019600* (GenSAS v6.0)	148,681 – 152,276	3,595 (3,315 w/o introns)	1,104

Table S4. Primers and PCR conditions used in this study.

Target	FW primer[a] (5' → 3')	RV primer[a] (5' → 3')	T_{ann}	Extension time
herA[b]	*atcg*GCGGCCGCatgtcctccatcgctgatacgca	*atcg*TTAATTAActagagactacgtgaaaatcgtaacaaggagc	71 ˚C	3 min 30 sec
herB	*atcg*GCGGCCGCatgccgattcccgtgaatactttcg	*catg*TTAATTAActactgcagtgcaccaacactcc	70 ˚C	2 min
g074890	*ctga*GCGGCCGCatggccaacgagaagacgcc	*atcg*TTAATTAAtcatacaacaatgctaagccggcc	70 ˚C	35 sec

a. 4-bp cleavage overhangs are italicized; recognition sites for restriction enzymes NotI (FW) and PacI (RV) are capitalized.

b. *herA* was amplified based on the sequence of gene 34 as predicted by fungiSMASH, which stretches 155 bp further than the reannotated *g019550* (see table S1).

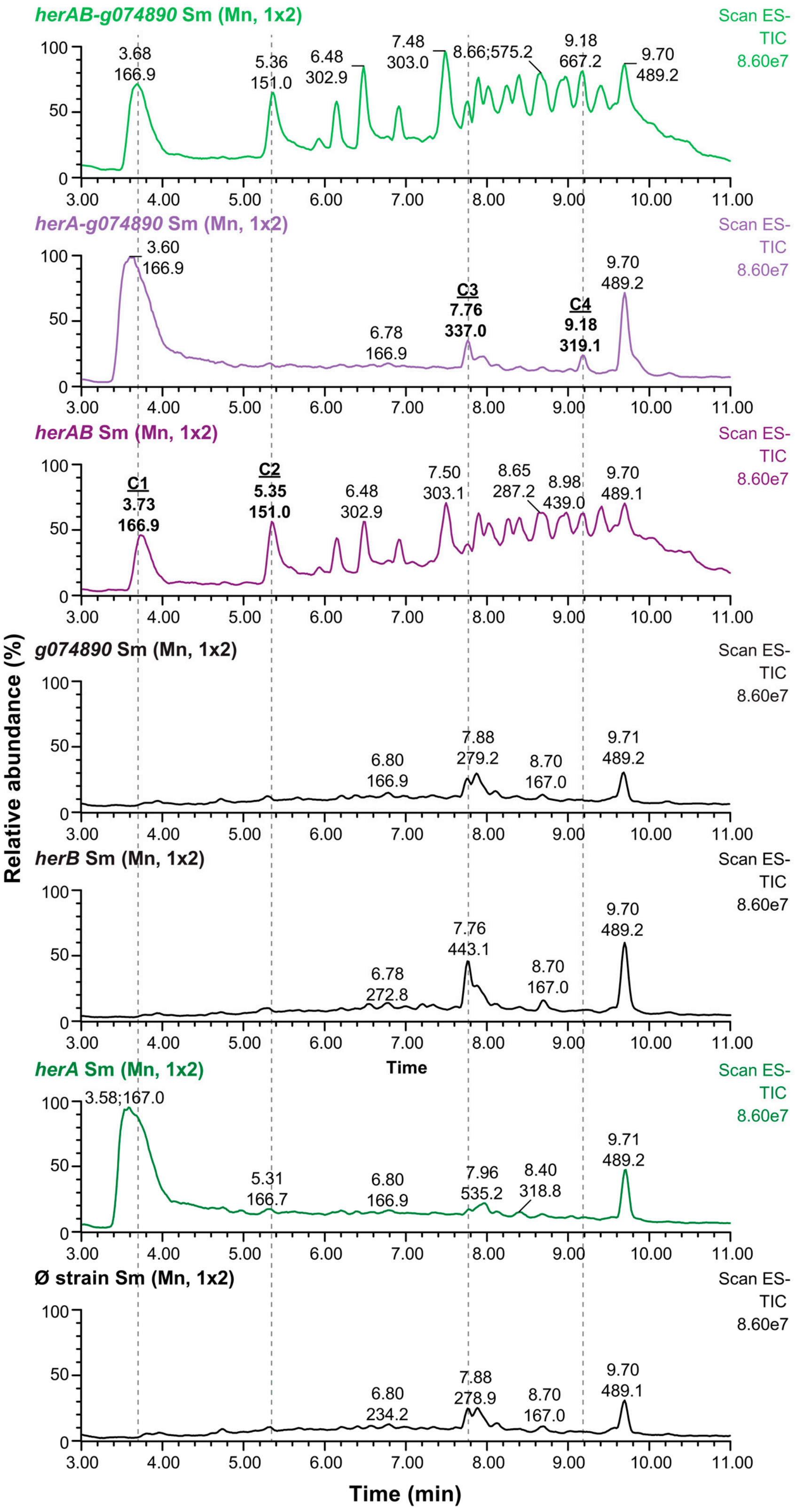
herAB-g074890 Sm (Mn, 1x2)
Scan ES- TIC 8.60e7
3.68 166.9
5.36 151.0
6.48 302.9
7.48 303.0
8.66;575.2
9.18 667.2
9.70 489.2
herA-g074890 Sm (Mn, 1x2)
Scan ES- TIC 8.60e7
3.60 166.9
6.78 166.9
C3 7.76 337.0
C4 9.18 319.1
9.70 489.2
herAB Sm (Mn, 1x2)
Scan ES- TIC 8.60e7
C1 3.73 166.9
C2 5.35 151.0
6.48 302.9
7.50 303.1
8.65 287.2
8.98 439.0
9.70 489.1
g074890 Sm (Mn, 1x2)
Scan ES- TIC 8.60e7
6.80 166.9
7.88 279.2
8.70 167.0
9.71 489.2
herB Sm (Mn, 1x2)
Scan ES- TIC 8.60e7
6.78 272.8
7.76 443.1
8.70 167.0
9.70 489.2
Time
herA Sm (Mn, 1x2)
Scan ES- TIC 8.60e7
3.58;167.0
5.31 166.7
6.80 166.9
7.96 535.2
8.40 318.8
9.71 489.2
Ø strain Sm (Mn, 1x2)
Scan ES- TIC 8.60e7
6.80 234.2
7.88 278.9
8.70 167.0
9.70 489.1
Relative abundance (%)
Time (min)

Figure S1. Total ion chromatograms of fungal extracts from *A. oryzae* strains. Extract of control strain *A. oryzae* NSAR1 is at the bottom. Despite differences in signal intensity for control strains *herB* and *g074890,* all the peaks are attributable to the background of the empty strain transformed with pTYsC or pTYadeA vectors. Compounds 1 to 4 are highlighted as in Figure 1. Peaks are annotated automatically by the proprietary software MassLynx v4.2 (Waters) based on the retention time at the highest point of the peak, and on the *m/z* value of the most abundant ion at that specific t_R. Chromatograms are smoothed (window size (scans): ± 2; iterations: 1; method: mean) for visualization purposes.

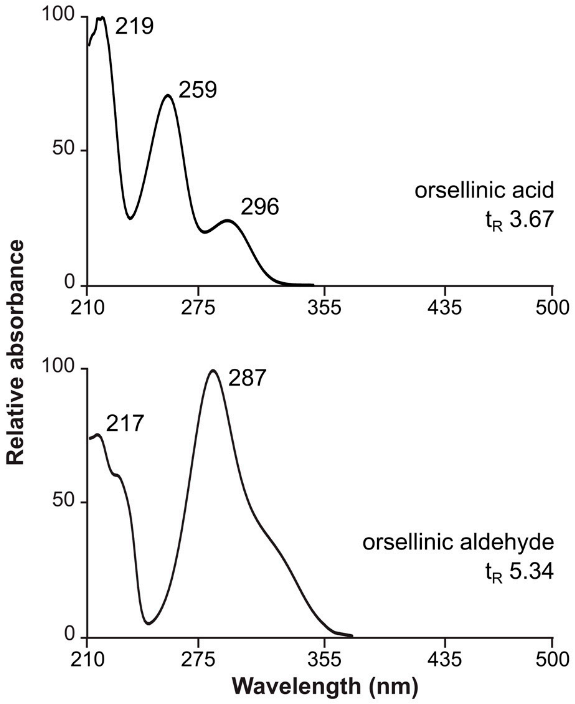

Figure S2. UV-Vis spectra of orsellinic acid and orsellinic aldehyde in extract of *herAB* expression strain. Peaks are annotated automatically by the proprietary software MassLynx v4.2 (Waters) based on wavelength at the highest point of the peak.

a

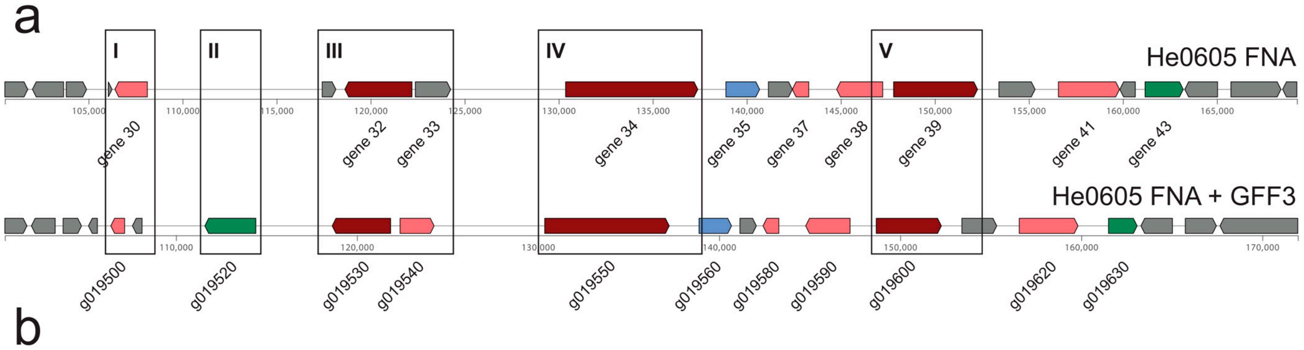

b

I - gene 30 flavoprotein + phocein-family domain (1,728 bp) → **flavoprotein g019500 (765 bp)** + phocein-family unknown g019510 (535 bp)

II - previously unannotated gene now identified: **gene 019520 (2,810 bp) putative fungal specific Zn(2)-Cys(6) transcription factor**

III - gene 32 NRPS-like (3,571 bp) → **g019530 (3,228 bp)**; gene 33 unknown (1,881 bp) → **aldehyde dehydrogenase g019540 (1,881 bp)**

IV - gene 34 PKS type I (7,033 bp) → **g019600 (6,878 bp)**

V - gene 39 NRPS-like (4,463 bp) → **g019600 (3,595 bp)**

Figure S3. Hericenones BGC prediction by fungiSMASH. (a) visual representation of hericenone BGC (HE-BGC4.1) as predicted by fungiSMASH v7.0 [1] using only the genomic FASTA file as input or genomic FASTA file plus annotation file generated with the web-tool GenSAS v6.0 [2]. Several differences are noted, in particular within five regions (I-V) where genes with a putative function are predicted. **(b)** key differences with respect to gene annotation/prediction in the highlighted genomic regions.

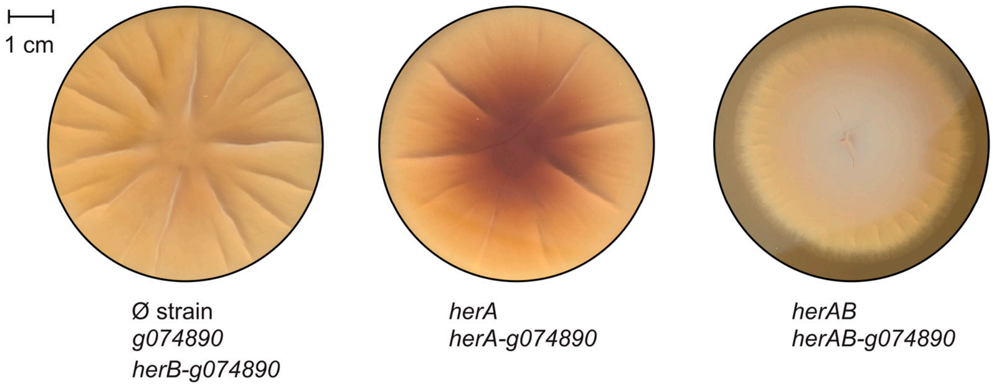

Figure S4. Morphology of *A. oryzae* strains (reverse view). Overexpression of *herA* leads to secretion of a dark brown pigment in the agar, likely a byproduct of orsellinic acid biosynthesis and/or degradation. Growth and appearance remain otherwise unchanged. Co-expression of *herA* and *herB* results in slower growth, and the mycelium appears velvety white (particularly on the agar side) and noticeably more compact.

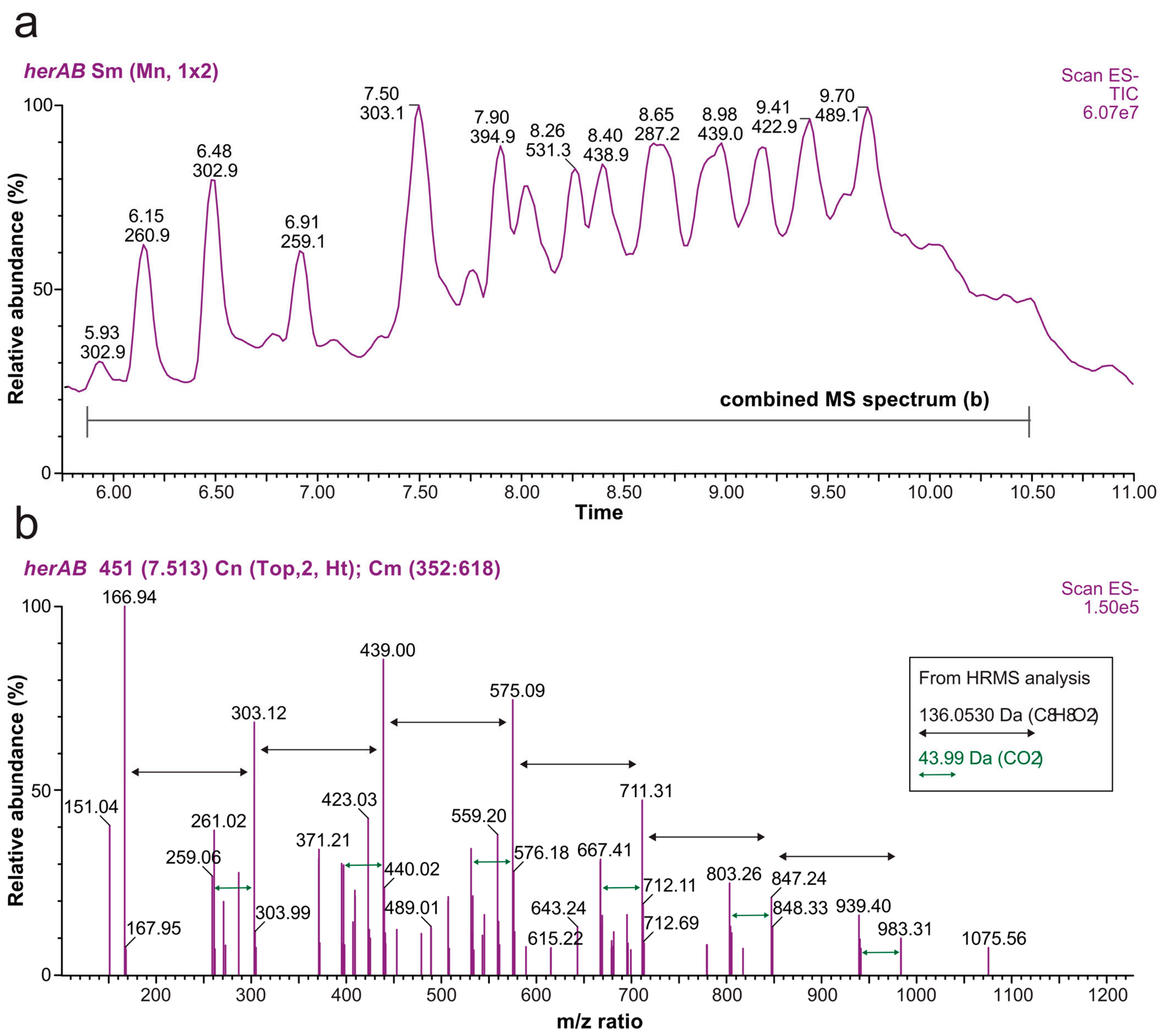

Figure S5. LC-MS analysis of unidentified compounds from fungal extract from *herAB* overexpression strain. (a) Chromatogram of fungal extract displaying TIC at t_R range ~ 5.75 – 11.00 min, where the series of unknown peaks from the extract appears. **(b)** Analysis of the combined spectrum reveals that the most abundant ions detected show repeating units of 136 and 44 (136.0530 and 43.99, respectively, from corresponding HRMS analysis of select peaks). These are consistent with repeated additions of $C_8H_8O_2$ units—indicative of polymerization—and corresponding decarboxylation.

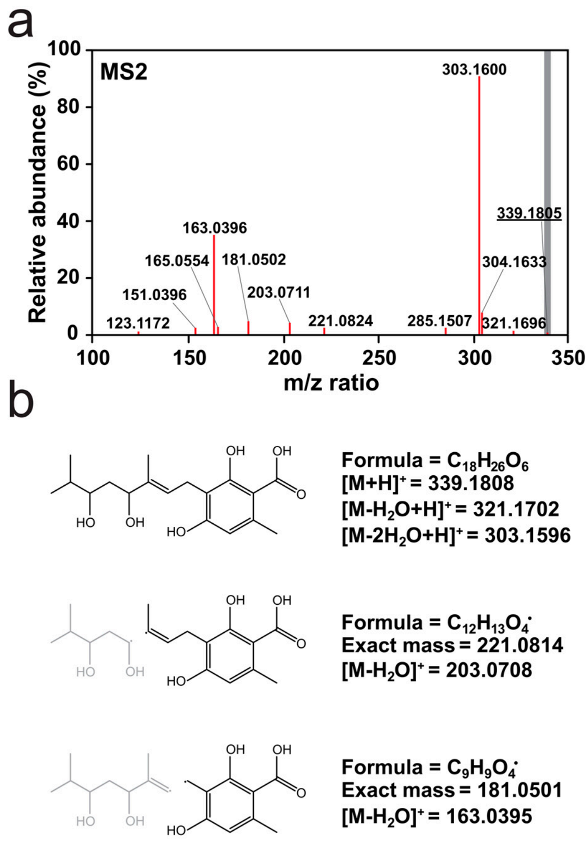

Figure S6. HRMS-MS-based analysis of compound 3. (a) MS2 spectrum of compound 3, showing two major fragments at *m/z* 303.1600 and 163.0396. **(b)** Proposed chemical structure of compound 3 and its fragments (in black) based on exact mass calculations and adduct formation. The analysis suggests that C3 might be a geranylated variant of orsellinic acid with two hydroxylations on the geranyl moiety. Chemical purification and NMR are required for confirmation.

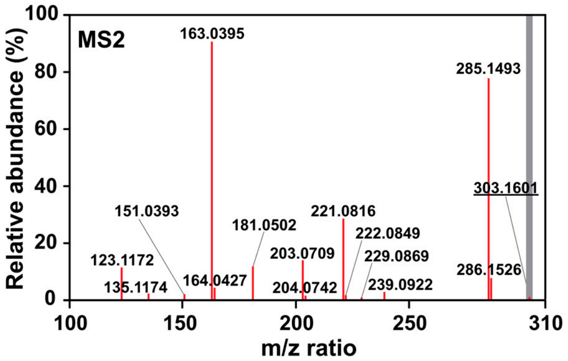

Figure S7. MS2 spectrum of compound 4. The spectrum shows similarity with that of compound 3, but no deductions could be made based on the *m/z* value of 303.1601, for the precursor ion. In low-resolution MS analysis (negative mode), we detected a *m/z* value of 319 $[M-H]^-$, possibly indicating a mono-hydroxylated orsellinic acid variant. We could not detect the corresponding *m/z* value of 321 $[M+H]^+$ in positive mode, neither with low-resolution MS nor with HRMS, thus we refrain from further propositions.

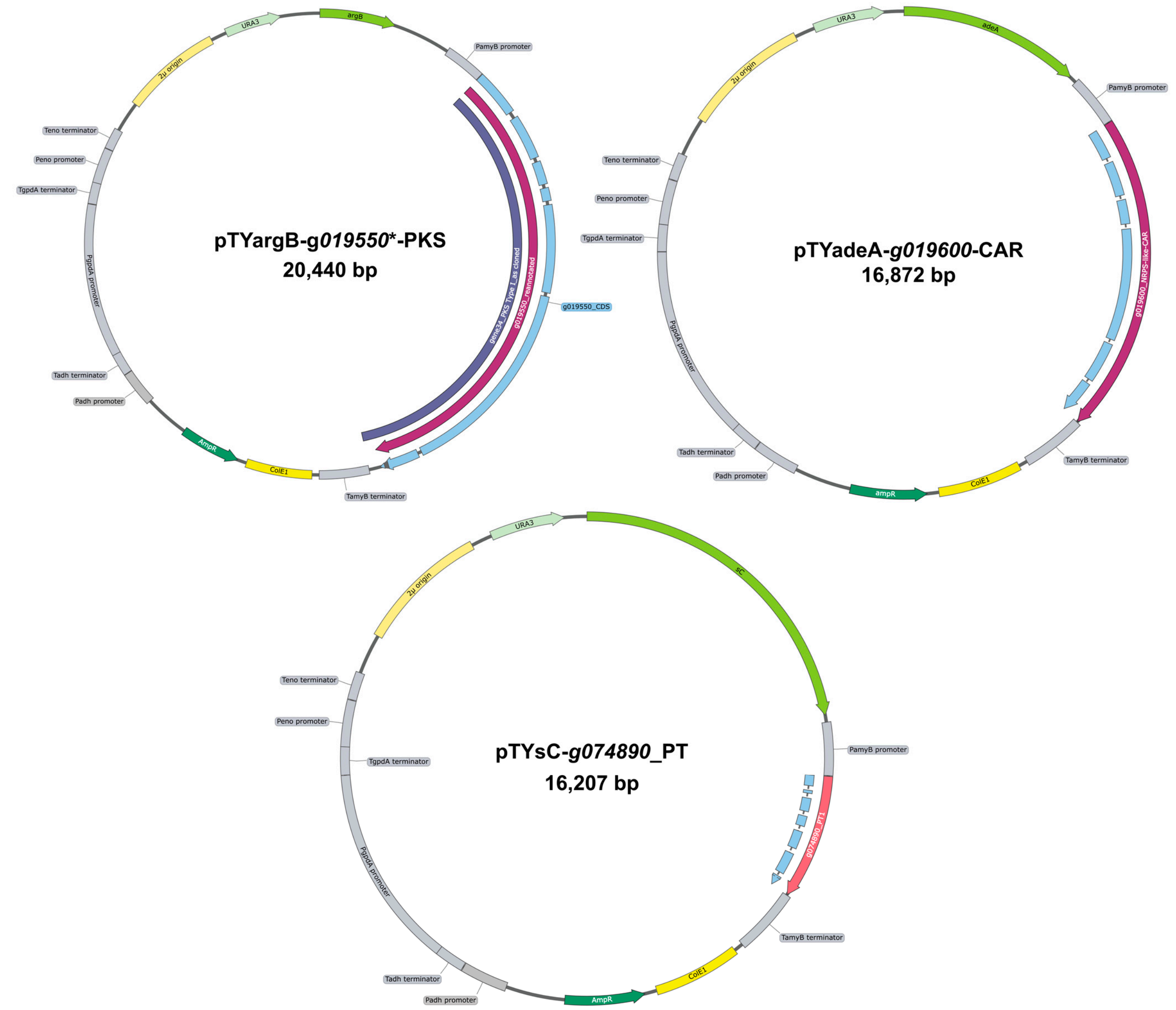

Figure S8. Maps of *herA*, *herB*, and *g074890* overexpression plasmids. The corresponding features are displayed both as full genes (continuous arrows) or as intron-free coding sequences (interrupted arrows). *NB: *g019550* was cloned prior re-annotation with GenSAS v6.0, and therefore contains an extra stretch of nt at the 3’, highlighted in Table S1. The full cloned features is annotated as a box, whereas the re-annotated gene and corresponding intron-free CDS as arrows. Plasmid maps generated with SnapGene software (www.snapgene.com).

Supplementary references

1. Blin K, Shaw S, Augustijn HE, Reitz ZL, Biermann F, Alanjary M, et al. antiSMASH 7.0: new and improved predictions for detection, regulation, chemical structures and visualisation. Nucleic Acids Res. 2023;51(W1):W46–50. https://doi.org/10.1093/nar/gkad344

2. Humann JL, Lee T, Ficklin S, Main D. Structural and Functional Annotation of Eukaryotic Genomes with GenSAS. In: Kollmar, M (eds) Gene Prediction Methods in Molecular Biology, vol 1962 Humana, New York, NY. 2019. p. 29–51. https://doi.org/10.1007/978-1-4939-9173-0_3
